# Expansion of the (BB) 〉metallacycle with coinage metal cations: formation of B–M–Ru–B (M = Cu, Ag, Au) dimetalacyclodiboryls†
†Electronic supplementary information (ESI) available: Experimental details and spectroscopic characterization. CCDC 1816708–1816710. For ESI and crystallographic data in CIF or other electronic format see DOI: 10.1039/c8sc00190a


**DOI:** 10.1039/c8sc00190a

**Published:** 2018-02-05

**Authors:** Bennett J. Eleazer, Mark D. Smith, Alexey A. Popov, Dmitry V. Peryshkov

**Affiliations:** a Department of Chemistry and Biochemistry , University of South Carolina , 631 Sumter St. , Columbia , South Carolina 29208 , USA . Email: peryshkov@sc.edu; b Leibniz Institute for Solid State and Materials Research , Helmholtzstrasse 20 , 01069 Dresden , Germany . Email: A.Popov@ifw-dresden.de

## Abstract

In this work, we introduce a novel approach for the selective assembly of heterometallic complexes by unprecedented coordination of coinage metal cations to strained single ruthenium–boron bonds on a surface of icosahedral boron clusters. M(i) cations (M = Cu, Ag, and Au) insert into B–Ru bonds of the (BB)–carboryne complex of ruthenium with the formation of four-membered B–M–Ru–B metalacycles. Results of theoretical calculations suggest that bonding within these metalacycles can be best described as unusual three-center-two-electron B–M···Ru interactions that are isolobal to B–H···Ru borane coordination for M = Cu and Ag, or the pairs of two-center-two electron B–Au and Au–Ru interactions for M = Au. These transformations comprise the first synthetic route to exohedral coinage metal boryl complexes of icosahedral *closo*-{C_2_B_10_} clusters, which feature short Cu–B (2.029(2) Å) and Ag–B (2.182(3) Å) bonds and the shortest Au–B bond (2.027(2) Å) reported to date. The reported heterometallic complexes contain Cu(i) and Au(i) centers in uncharacteristic square-planar coordination environments. These findings pave the way to rational construction of a broader class of multimetallic architectures featuring M–B bonds.

## Abstract 2

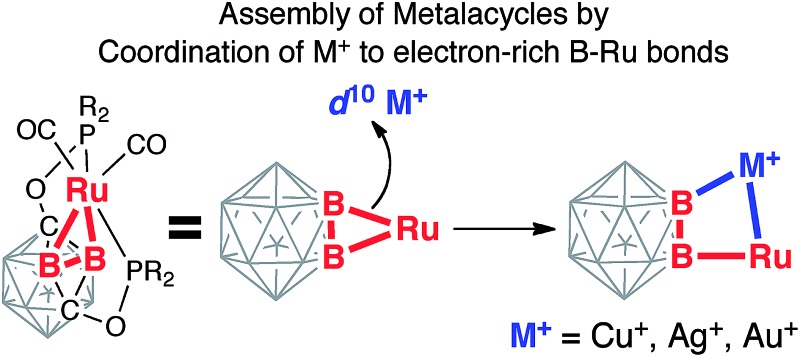
Assembly of bimetallic complexes on boron clusters by coordination of coinage metal cations to Ru–B single bonds.

## Introduction

Synthesis and reactivity of bimetallic complexes containing late transition metals and coinage metals have attracted considerable attention due to the discovered cooperative reactivity in cross-coupling reactions, transmetallation processes, and the relevance to heterogeneous catalysis.^1^–^7^ One fruitful synthetic strategy for the formation of these complexes is the reaction of a nucleophilic anionic metal complex with an electrophilic coinage metal cation source.^8^–^12^ Another general approach to heterobimetallic complexes includes the use of multifunctional ligands containing several types of donor sites, which, upon stepwise selective metalation enforce metal–metal interactions.^13^–^15^ A distinct route to heterobimetallic complexes is also the coordination of an electrophilic metal moiety to metal alkylidene, alkylidyne, and silylene groups.^16^–^19^ The isolobal analogy concept has been recently used to extend this reactivity pattern to metal borylene complexes, which have been found to coordinate electrophilic metal cations to manganese–boron double bonds.^20^

Transition metal boryl complexes have been extensively studied because of both fundamental and applied reactivity potential. Success in metal-catalyzed borylation of a variety of organic substrates led to the enormous growth in studies of structure, bonding, and reactivity of complexes, containing metal–boron bonds.^21^–^28^ One of the subsets of these compounds are B-carboranyl complexes, containing exohedral metal–boron bonds on the surface of heteroborane cages, such as *closo*-C_2_B_10_H_12_.^29^–^32^ In contrast to many transition metal boryl complexes, the metal–boron bonds in B-carboranyl complexes are often exceedingly stable due in part to the high degree of steric shielding by the icosahedral borane cage. The exohedral metal–boron bonding in icosahedral B-carboranyls is dominated by two-center-two-electron (2c-2e) σ-type interactions because p-orbitals of boron atoms are involved in delocalized cluster bonding, although there is growing evidence of the possibility of exohedral π-bonding of cage borons with non-metals.^33^,^34^ This sets B-carboranyls apart from other metal boryl complexes, which may potentially exhibit some degree of π-type metal–boron interactions, and from cluster metalloboranes, which possess bonding analogous to that in cyclopentadienyl complexes with no distinct 2c-2e metal–boron bonds. This difference is often highlighted by regarding carboranes as three-dimensional aromatic analogs of arenes.^35^–^37^ Unusual electronic and steric properties of carboranes clusters led to their use in ligand design, catalysis, electrochemistry, polymer science, and photophysics.^38^–^53^

The exohedral metal center on a carborane cage can be simultaneously connected to two vertices giving rise to carborynes, which are inorganic analogs of benzynes.^54^–^58^ We recently reported the synthesis of the first BB-carboryne complex, which features two boron atoms of the carborane cage connected to a single ruthenium metal center.^59^ The resulting (BB) <svg xmlns="http://www.w3.org/2000/svg" version="1.0" width="16.000000pt" height="16.000000pt" viewBox="0 0 16.000000 16.000000" preserveAspectRatio="xMidYMid meet"><metadata> Created by potrace 1.16, written by Peter Selinger 2001-2019 </metadata><g transform="translate(1.000000,15.000000) scale(0.005147,-0.005147)" fill="currentColor" stroke="none"><path d="M0 2080 l0 -80 80 0 80 0 0 -40 0 -40 160 0 160 0 0 -40 0 -40 160 0 160 0 0 -40 0 -40 160 0 160 0 0 -40 0 -40 160 0 160 0 0 -40 0 -40 160 0 160 0 0 -40 0 -40 160 0 160 0 0 -40 0 -40 160 0 160 0 0 -40 0 -40 160 0 160 0 0 80 0 80 -160 0 -160 0 0 40 0 40 -160 0 -160 0 0 40 0 40 -160 0 -160 0 0 40 0 40 -160 0 -160 0 0 40 0 40 -160 0 -160 0 0 40 0 40 -160 0 -160 0 0 40 0 40 -160 0 -160 0 0 40 0 40 -160 0 -160 0 0 40 0 40 -80 0 -80 0 0 -80z M2400 1000 l0 -40 -160 0 -160 0 0 -40 0 -40 -160 0 -160 0 0 -40 0 -40 -160 0 -160 0 0 -40 0 -40 -160 0 -160 0 0 -40 0 -40 -160 0 -160 0 0 -40 0 -40 -160 0 -160 0 0 -40 0 -40 -160 0 -160 0 0 -40 0 -40 -80 0 -80 0 0 -80 0 -80 80 0 80 0 0 40 0 40 160 0 160 0 0 40 0 40 160 0 160 0 0 40 0 40 160 0 160 0 0 40 0 40 160 0 160 0 0 40 0 40 160 0 160 0 0 40 0 40 160 0 160 0 0 40 0 40 160 0 160 0 0 40 0 40 160 0 160 0 0 80 0 80 -160 0 -160 0 0 -40z"/></g></svg> Ru metalacycle can be described as a metalacycloboropropane with two highly strained 2c-2e B–Ru σ bonds. The significant distortion of the exohedral bonds of the carborane cage resulted in enhanced reactivity associated with these bent B–Ru bonds, which themselves served as nucleophilic reaction centers with organic substrates, exhibiting metal–ligand cooperative activity. We conjectured that the bonding pair of the distorted electron-rich B–Ru bond in the BB-carboryne metallacycle could be accessible for interaction with inorganic electrophiles such as coinage metal cations.

**Scheme 1:**
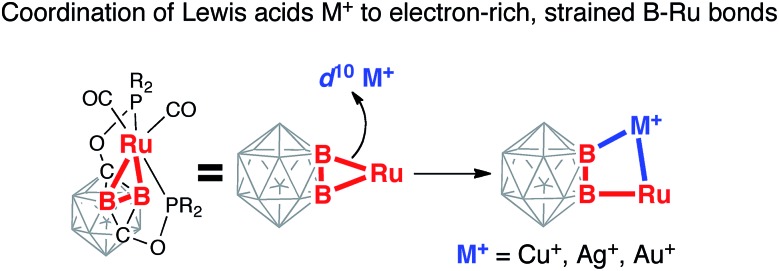
Coordination of coinage metal cations to strained B–Ru single bonds of the (BB)-carboryne complex with the formation of B–M–Ru–B metalacycles.

Here we report the first examples of coordination of Lewis acidic Cu(i), Ag(i), and Au(i) to a single Ru–B metal–boryl bond and the formation of the heterobimetallic complexes featuring unique B–M–Ru–B bridging interactions (Scheme 1). This approach led to the synthesis of the first examples of *closo*-carborane clusters containing exohedral B–Cu, B–Ag, and B–Au bonds.

## Results and discussion

### Synthesis and structures of the bimetallic complexes

**Fig. 1:**
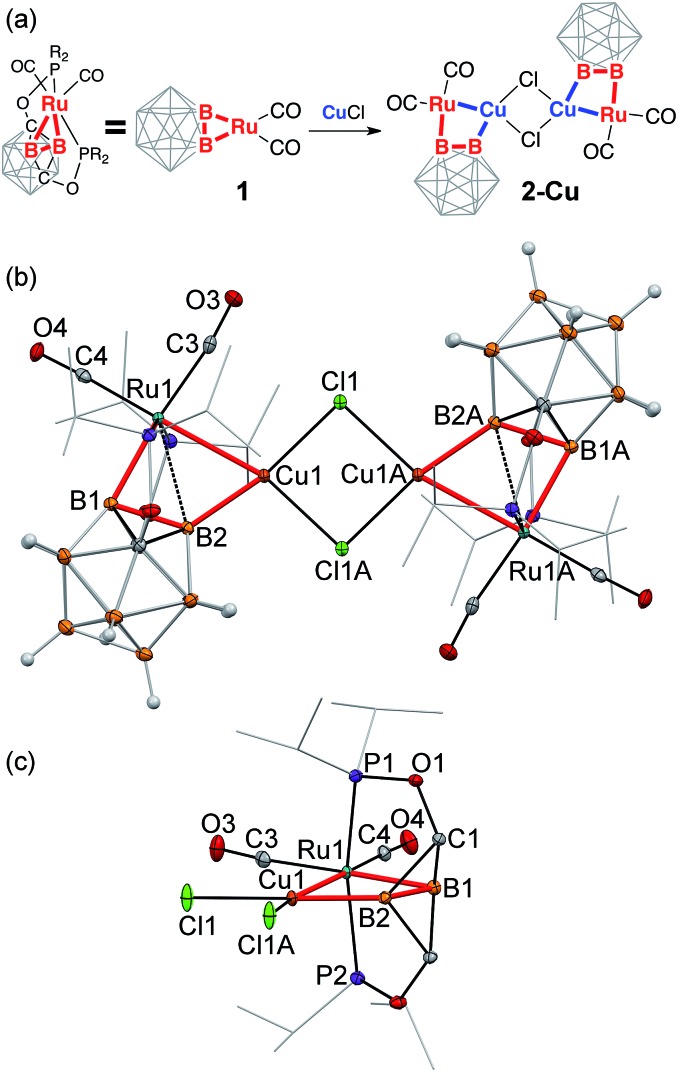
(a) Synthesis of [(POBBOP)(Ru)(CO)_2_(Cu)(Cl)]_2_ (**2-Cu**, POBBOP = 1,7-OP(i-Pr)_2_-2,6-dehydro-*m*-carborane). (b, c) Displacement ellipsoid plot (50% probability) of the [(POBBOP)Ru(CO)_2_(Cu)(Cl)]_2_ complex (**2-Cu**). (b) A general view (c) a coordination environment of Ru and Cu centers. Note the distorted square-planar ligand arrangement around Cu(i). Atoms belonging to isopropyl groups of the ligand arms are omitted for clarity.

**Fig. 2:**
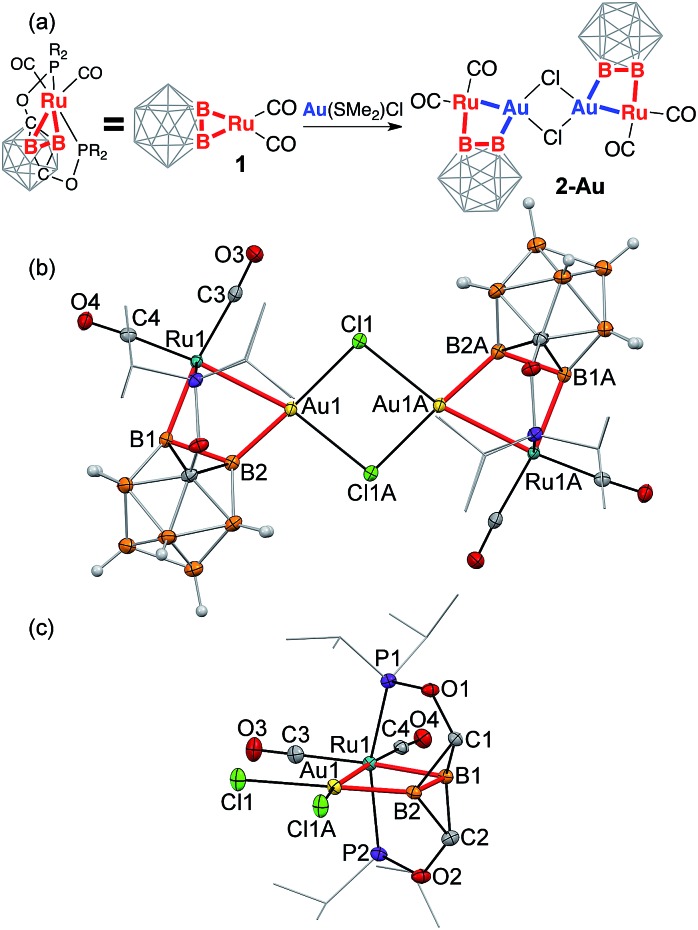
(a) Synthesis of [(POBBOP)Ru(CO)_2_(Au)(Cl)]_2_ (**2-Au**). (b, c) Displacement ellipsoid plot (50% probability) of the [(POBBOP)Ru(CO)_2_(Au)(Cl)]_2_ complex. (b) A general view (c) a coordination environment of Ru and Au centers. Note the distorted square-planar ligand arrangement around Au(i). Atoms belonging to isopropyl groups of the ligand arms are omitted for clarity.

**Fig. 3:**
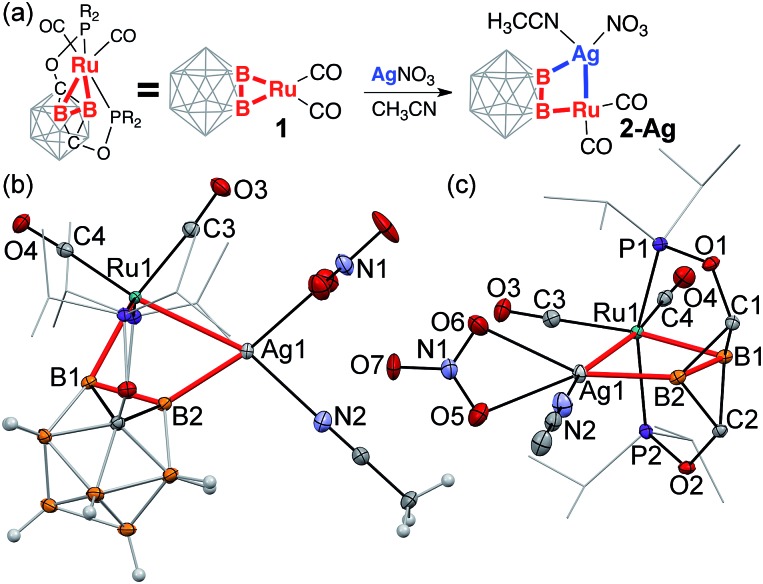
(a) Synthesis of (POBBOP)(Ru)(CO)_2_(Ag)(CH_3_CN)(NO_3_) (**2-Ag**). (b, c) Displacement ellipsoid plot (50% probability) of the (POBBOP)(Ru)(CO)_2_(Ag)(CH_3_CN)(NO_3_) complex (**2-Ag**). (b) A general view (c) a coordination environment of Ru and Ag centers. Atoms belonging to isopropyl groups of the ligand arms are omitted for clarity.

Reaction of the (POBBOP)Ru(CO)_2_ carboryne complex **1** (POBBOP = 1,7-OP(i-Pr)_2_-*m*-2,6-dehydrocarborane)^59^ with CuCl, AgNO_3_, and Au(SMe_2_)Cl in 1 : 1 ratio led to the selective formation and isolation of complexes **2-Cu**, **2-Ag**, and **2-Au**, respectively. The ^31^P and ^11^B NMR spectra of crude reaction mixtures reflected clean transformation of **1** to new products, which possessed lower symmetry of a boron cage according to ^11^B NMR spectra, consistent with interaction of coinage metal cations with one of the B–Ru bonds of the parent carboryne **1**. The ^11^B and ^11^B{^1^H} spectra of products contained pairs of signals corresponding to two different metalated boron atoms of the icosahedral cage, indicating the persistence of such interactions in solution. The complexes **2-Cu**, **2-Ag**, and **2-Au** were isolated in 70–80% yields and were found to be moderately stable in air in the solid state in the absence of light. The single crystal X-ray diffraction study revealed the molecular structures of **2-Cu**, **2-Ag**, and **2-Au** (Fig. 1–3 and S21–S23 in ESI†). In all three complexes, a coinage metal cation was found in the bridging position between the boron atom and the ruthenium metal center with the second B–Ru bond of the parent carboryne remaining intact. Two carbonyl ligands remained bound to the ruthenium center. The summary of the selected bond distances and angles is given in Table 1.

**Table 1:** Selected interatomic distances (Å) and angles (°) in **2-M** (M = Cu, Ag, and Au) complexes

	**2-Cu**	**2-Ag**	**2-Au**
Ru1–B1	2.135(2)	2.143(3)	2.133(2)
Ru1–B2	2.475(2)	2.444(3)	2.723(3)
Ru1–M1	2.630(1)	2.750(1)	2.682(1)
M1–B2	2.029(2)	2.182(3)	2.027(2)
B2–B1–Ru1^*a*^	78.5(1)	77.4(1)	87.9(1)
B2–M1–Cl1	170.2(1)	—	177.5(1)
Ru1–M1–Cl1A	164.6(1)	—	167.5(1)
Ru1–M1–B2	62.6(1)	58.1(1)	69.1(1)
Ru1–M1–Cl1	107.7(1)	—	108.4(1)

^*a*^For comparison, the unstrained B2–B1–H1 angle in the ligand precursor POBOP-H is 116.1(1)°.

The complex **2-Cu** crystallized as a chloride-bridged dimer from an acetonitrile/hexanes mixture (Fig. 1). The copper cation is coordinated to one of the former Ru–B single bonds of carboryne **1**. Remarkably, the Cu1–B2 bond length in **2-Cu** is 2.029(2) Å, which is only slightly longer than 2c-2e Cu–B bonds in the recently reported Cu(i) boryl complexes (1.980(2)–2.002(3) Å).^60^–^66^ This bond length is significantly shorter than the Cu–B distance in anionic metallacarborane–copper complexes (2.111(8)–2.208(2) Å) where a boron atom of the open face of a carbollide ligand bridges two metal centers.^67^–^76^ The Cu1–Ru1 distance in **2-Cu** is 2.630(1) Å, which is longer than Cu–Ru bonds in bimetallic complexes (2.439(1)–2.552(7) Å)^77^ and comparable to the corresponding distances in multimetallic copper–ruthenium clusters (2.552(2)–2.939(2) Å).^78^–^87^ The Ru1–B1 bond length of 2.135(2) Å is within the typical range for exohedral 2c-2e Ru–B bonds of boron clusters.^59^,^88^–^90^ In contrast, the Ru1–B2 distance of 2.475(2) Å, which is out of the range for the direct 2c-2e boron–ruthenium bond, lies at the upper end of the characteristic range for boron cluster complexes with bridging 3c-2e B–H···Ru interactions (2.355(3)–2.462(3) Å).^91^–^95^ The Ru1–B1 bond is strained as indicated by the acute B2–B1–Ru1 angle of 78.5(1)°, which dramatically deviates from the analogous angle for the unstrained B–H bond in the ligand precursor POBOP-H (116.1(1)°).^96^ This distortion is likely caused by attractive Ru1···Cu1 and Ru1···B2 interactions that bring the ruthenium metal center closer to the B2–Cu1 bond thus causing the Ru1–B1 bond strain.

Two bridging chloride ligands (Cu–Cl distances are 2.321(1) Å and 2.329(1) Å) complete the coordination sphere of the copper center to four-coordinate planar geometry, which is uncharacteristic for Cu(i). The distortion from the planarity for the Cu(Cl)_2_(B)(Ru) unit is remarkably small with the *τ*_4_ value of 0.17 (*τ*_4_ = 0 for ideal square-planar arrangement, *τ*_4_ = 1 for tetrahedral geometry). To the best of our knowledge, this is one of the lowest values of the *τ*_4_ parameter for Cu(i) complexes reported to date.^97^–^101^

**2-Au** crystallized from an acetonitrile/hexanes mixture as a chloride-bridged dimer, with geometry analogous to **2-Cu** (Fig. 2). The Au(i) cation inserted into one of the B–Ru bonds of the carboryne complex. The Au1–B2 bond length is 2.027(2) Å, which is the shortest Au–B distance reported to date, as it is slightly shorter than 2c-2e gold–boron bonds in the previously disclosed gold boryl complexes (2.069(3)–2.144(4) Å).^61^,^62^,^102^ The Au1–Ru1 distance is 2.682(1) Å, which is comparable to the previously reported distances in bimetallic complexes (the lowest value reported is 2.694(1) Å).^103^ In contrast to **2-Cu**, the relatively long Ru1–B2 distance of 2.723(3) Å implies no significant bonding while the Ru1–B1 bond length (2.133(2) Å) is within the typical range for ruthenium boryls.

Similarly to the copper coordination environment in **2-Cu**, the Au(Cl)_2_(B)(Ru) moiety in **2-Au** exhibits four-coordinate planar configuration that is unusual for Au(i)^104^–^106^ with slight distortion (*τ*_4_ = 0.11) as indicated by nearly linear Ru1–Au1–Cl1A and B2–Au1–Cl1 angles (167.5(1)° and 177.5(1)°, respectively).

The light-sensitive silver insertion product, **2-Ag**, crystallized from an acetonitrile/hexanes mixture as a monomeric acetonitrile adduct (Fig. 3). The Ag(i) cation inserted into one of the B–Ru bonds of the carboryne complex. The Ag1–B1 bond length is 2.182(3) Å, which is, to the best of our knowledge, the third example of the silver–boryl bond and this distance is comparable to those in two previously reported silver boryl complexes (2.118(2) Å and 2.122(4) Å).^61^

The Ru1–Ag1 distance is 2.750(1) Å, which is longer than the previously reported distances in unsupported bimetallic complexes (2.608(1)–2.709(1) Å)^9^,^107^ but shorter than in multimetallic clusters (the smallest value being 2.767(1) Å).^108^ Interestingly, the Ag–Ru distance in the ruthenium silylene complex that binds Ag^+^ across the ruthenium–silicon double bond is 2.681(1) Å.^19^ The Ru1–B1 distance is typical at 2.143(3) Å while the Ru1···B2 distance is relatively short at 2.444(3) Å indicating significant interaction similarly to that in **2-Cu**.

Trends in values of stretching frequencies of carbonyl ligands of the ruthenium center can provide an insight into changes of its electronic structure upon conversion of **1** to **2-M** complexes. The values of *ν*(CO) = 2010 and 1958 cm^–1^ (*ν*(CO)_average_ = 1984 cm^–1^) for the BB-carboryne complex **1** can be compared to the corresponding parameters in coinage metal insertion products (see ESI† for FTIR spectra). The *ν*(CO)_average_ values for these complexes are 1995 cm^–1^ for **2-Cu**, 2002 cm^–1^ for **2-Au**, and 2017 cm^–1^ for **2-Ag**. Thus, in all three cases, the *ν*(CO)_average_ increased reflecting coordination of Lewis acidic cations to one of Ru–B bonds in **1**.

The comparison of ^11^B NMR spectra of **1** and **2-M** in C_6_D_6_ showed changes of chemical shifts of metalated boron atoms upon coordination of coinage metals. The starting complex **1** exhibited a signal for the (BB) <svg xmlns="http://www.w3.org/2000/svg" version="1.0" width="16.000000pt" height="16.000000pt" viewBox="0 0 16.000000 16.000000" preserveAspectRatio="xMidYMid meet"><metadata> Created by potrace 1.16, written by Peter Selinger 2001-2019 </metadata><g transform="translate(1.000000,15.000000) scale(0.005147,-0.005147)" fill="currentColor" stroke="none"><path d="M0 2080 l0 -80 80 0 80 0 0 -40 0 -40 160 0 160 0 0 -40 0 -40 160 0 160 0 0 -40 0 -40 160 0 160 0 0 -40 0 -40 160 0 160 0 0 -40 0 -40 160 0 160 0 0 -40 0 -40 160 0 160 0 0 -40 0 -40 160 0 160 0 0 -40 0 -40 160 0 160 0 0 80 0 80 -160 0 -160 0 0 40 0 40 -160 0 -160 0 0 40 0 40 -160 0 -160 0 0 40 0 40 -160 0 -160 0 0 40 0 40 -160 0 -160 0 0 40 0 40 -160 0 -160 0 0 40 0 40 -160 0 -160 0 0 40 0 40 -160 0 -160 0 0 40 0 40 -80 0 -80 0 0 -80z M2400 1000 l0 -40 -160 0 -160 0 0 -40 0 -40 -160 0 -160 0 0 -40 0 -40 -160 0 -160 0 0 -40 0 -40 -160 0 -160 0 0 -40 0 -40 -160 0 -160 0 0 -40 0 -40 -160 0 -160 0 0 -40 0 -40 -160 0 -160 0 0 -40 0 -40 -80 0 -80 0 0 -80 0 -80 80 0 80 0 0 40 0 40 160 0 160 0 0 40 0 40 160 0 160 0 0 40 0 40 160 0 160 0 0 40 0 40 160 0 160 0 0 40 0 40 160 0 160 0 0 40 0 40 160 0 160 0 0 40 0 40 160 0 160 0 0 40 0 40 160 0 160 0 0 80 0 80 -160 0 -160 0 0 -40z"/></g></svg> Ru metallacycle at –2.8 ppm while the corresponding signals for the boron atom of the B–Ru bond are at –1.5 ppm for **2-Cu**, +2.1 ppm for **2-Au**, and –2.7 ppm for **2-Ag**. The signals of boron atoms of B–M bonds are at –10.3 ppm for **2-Cu**, –9.7 ppm for **2-Au**, and –11.9 for **2-Ag**. The ^31^P spectra of **2-M** complexes are similar with a signal in the 204–205 ppm range (the starting complex **1** exhibited a signal in the ^31^P NMR spectrum at 217 ppm).

### Theoretical calculations

**Chart 1:**
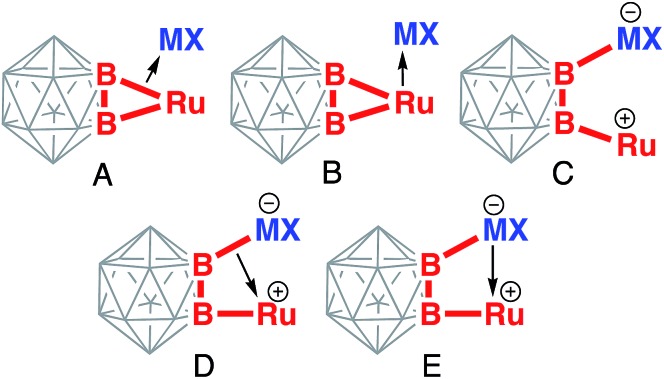
Possible bonding descriptions of the coordination of coinage metal cations M^+^ to the (BB) <svg xmlns="http://www.w3.org/2000/svg" version="1.0" width="16.000000pt" height="16.000000pt" viewBox="0 0 16.000000 16.000000" preserveAspectRatio="xMidYMid meet"><metadata> Created by potrace 1.16, written by Peter Selinger 2001-2019 </metadata><g transform="translate(1.000000,15.000000) scale(0.005147,-0.005147)" fill="currentColor" stroke="none"><path d="M0 2080 l0 -80 80 0 80 0 0 -40 0 -40 160 0 160 0 0 -40 0 -40 160 0 160 0 0 -40 0 -40 160 0 160 0 0 -40 0 -40 160 0 160 0 0 -40 0 -40 160 0 160 0 0 -40 0 -40 160 0 160 0 0 -40 0 -40 160 0 160 0 0 -40 0 -40 160 0 160 0 0 80 0 80 -160 0 -160 0 0 40 0 40 -160 0 -160 0 0 40 0 40 -160 0 -160 0 0 40 0 40 -160 0 -160 0 0 40 0 40 -160 0 -160 0 0 40 0 40 -160 0 -160 0 0 40 0 40 -160 0 -160 0 0 40 0 40 -160 0 -160 0 0 40 0 40 -80 0 -80 0 0 -80z M2400 1000 l0 -40 -160 0 -160 0 0 -40 0 -40 -160 0 -160 0 0 -40 0 -40 -160 0 -160 0 0 -40 0 -40 -160 0 -160 0 0 -40 0 -40 -160 0 -160 0 0 -40 0 -40 -160 0 -160 0 0 -40 0 -40 -160 0 -160 0 0 -40 0 -40 -80 0 -80 0 0 -80 0 -80 80 0 80 0 0 40 0 40 160 0 160 0 0 40 0 40 160 0 160 0 0 40 0 40 160 0 160 0 0 40 0 40 160 0 160 0 0 40 0 40 160 0 160 0 0 40 0 40 160 0 160 0 0 40 0 40 160 0 160 0 0 40 0 40 160 0 160 0 0 80 0 80 -160 0 -160 0 0 -40z"/></g></svg> Ru metalacycle.

Several resonance structures can be considered to describe bonding in B–Ru–M–B metallacycles in these complexes (Chart 1). The structure A is a side-on coordination of the coinage metal cation to the single Ru–B bond. In the structure B, the Lewis acidic cation is coordinated to the relatively basic Ru(ii) center making it heptacoordinate. In the structure C, coinage metal forms the 2c-2e electron bond to one of the boron atom of the cage, while the structure D features coordination of the B–M bond to the ruthenium center. The structure E is derived from the structure C by addition of the dative M–Ru interaction.

**Fig. 4:**
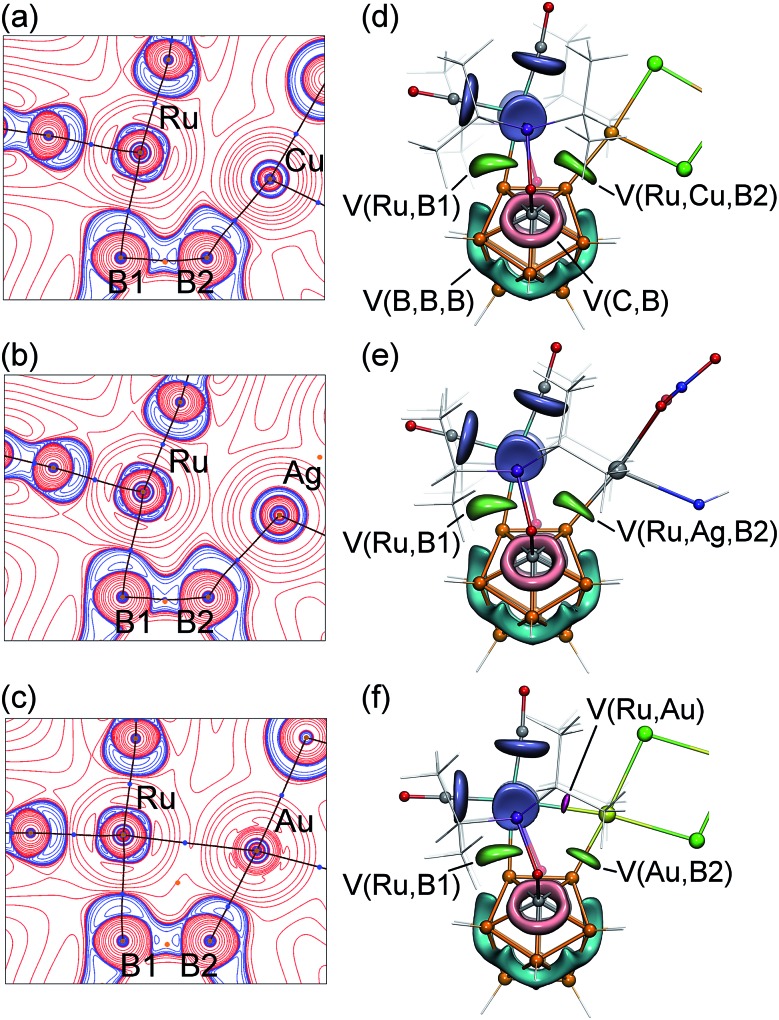
(a–c) The contour map of electron density Laplacian and molecular graphs (based on the QTAIM bond paths) in the (B1–B2–Ru1–M) plane for **2-Cu**, **2-Ag**, and **2-Au** (M = Cu, Ag, and Au; red curves denote electron density depletion, blue curves denote electron density concentration; blue dots are bond critical points). (d–f) ELF isosurfaces for selected valence basins for **2-Cu**, **2-Ag**, and **2-Au**. V(B,B) (cyan), V(B,C) (pink), V(Ru,P)/V(Ru,C) (dark-blue) basins are shown at the level *η* = 0.8; V(Ru,B1), V(Ru,Cu,B2), V(Ru,Ag,B2), and V(Au,B) basins are shown at *η* = 0.7 in light green color; the V(Ru,Au) basin is shown in red at *η* = 0.4. Other basins are omitted for clarity.

The presence of unique B–M–Ru–B metallacycles in the reported new complexes prompted us to examine the bonding situation in more detail using the analysis of the electron density in the framework of the quantum theory of atoms-in-molecules (QTAIM)^109^–^112^ as well as the analysis of the electron localization function (ELF)^113^,^114^ for the electron density computed at the PBE0/def-TZVP level with ZORA correction as implemented in Orca 3.0.3 suite.^115^,^116^ Results of the QTAIM topological analysis of the electron density are presented in Fig. 4a–c and Table 2.

**Table 2:** QTAIM delocalization indices (DI) in the B1–Ru–M–B2 fragments of **2-M** (M = Cu, Ag, Au) complexes

	**2-Cu**	**2-Ag**	**2-Au**
DI(Ru–B1)	0.73	0.74	0.75
DI(Ru–B2)	0.31	0.33	0.10
DI(Ru–M)	0.42	0.40	0.58
DI(M–B2)	0.66	0.63	0.81

Topological analysis revealed the presence of the Ru–B1 bond path in all complexes. The bonding between ruthenium and the boron atom B1 exhibits concentration of the electron density (blue contours) that is bent outward the Ru–B1 bond path, indicating the strain of this metal–boron bond. The Ru–B1 bonding in all three structures can be considered a 2c-2e bond with delocalization indices (DI, defined as the number of electron pairs shared between two atoms, which is considered as an analog of the bond order in QTAIM) of 0.73 for **2-Cu**, 0.74 for **2-Ag**, and 0.75 for **2-Au**. These values are also close to DI values corresponding to 2c-2e metalboron bonds in the starting carboryne complex **1** (DI = 0.69).

Coordination of the Ru–B2 bond to Cu, Ag, or Au substantially changes the bonding situation around it. Topological analysis revealed the second Ru–B2 bond path in none of the three complexes. Instead, the M–B2 bond path is developed in all complexes. The DI values of coinage metal–boron bonds are 0.66 for the Cu–B2 bond and 0.63 for the Ag–B2 bonds with the partial charge on B2 being similar to that of B1. Despite the absence of direct bond paths, the interactions between the ruthenium center and the vicinal boron atom B2 are significant in **2-Cu** and **2-Ag** as demonstrated by DI values of 0.31 (**2-Cu**) and 0.33 (**2-Ag**). The ruthenium–coinage metal interactions in these complexes do not have bond paths for **2-Cu** and **2-Ag.** The DI values of these interactions are 0.42 for **2-Cu**, 0.40 for **2-Ag**. Such an extensive “sharing” of electrons between Ru, B2, and Cu or Ag is indicative of 3-center-2-electron bonding (Chart 1, structure D, see also the ELF analysis below). These vicinal B–M···Ru interactions invoke an isolobal analogy to B–H···Ru interactions that are often strong for carboranyl complexes. For instance, the B2–H···Ru 3c-2e bond in the recently reported ruthenium carboranyl hydride complex with a similar geometry possessed a DI value of 0.23 for the Ru···B2 interaction.

The Ru–Au–B2 bonding situation is noticeably different. First, the QTAIM analysis revealed the presence of the Ru–Au bond path with a DI value of 0.58. The DI value of Au–B2 bond is 0.81, which is also higher than that for Cu–B2 and Ag–B2 bonds. At the same time, the Ru–B2 delocalization index in **2-Au** is reduced to 0.10. Based on these indicators, we can conclude that the 3-center bonding character in the **2-Au** complex is reduced in favor of more localized pairwise Ru–Au and Au–B2 interactions (Chart 1, structure E). Higher electronegativity of Au reveals itself in its more negative atomic charge (–0.05) when compared to the Cu and Ag charges of +0.4. This increase of the electron density on Au is largely achieved at the expense of the B2 atom, which atomic charge (+0.85) is considerably more positive than that of B1 (+0.58) in **2-Au** or the charge of the B2 atom in **2-Cu** and **2-Ag** complexes (+0.59 and +0.60, respectively). Note that the depletion of the electron density on B2 also results in the weakening of the B1–B2 bonding.

Electron localization function (ELF) is visualized by 3D representations (Fig. 4d–f), and its 2D maps near the Ru–M–B2 fragment (Fig. S20 in ESI†). Table S1 in ESI† summarizes attractor values (*η*_a_; the maximum ELF values for each basin) and basin populations (Ω, the number of electron per basin) for selected valence basins of interest. For **2-Cu** and **2-Ag**, the ELF analysis revealed the presence of one disynaptic valence basin V(Ru,B1) (which is similar to that in the starting complex **1**) and one trisynaptic V(Ru,Cu,B2) or V(Ru,Ag,B2) basin, respectively. Attractors of trisynaptic V(Ru,Cu,B) and V(Ru,Ag,B) basins are located close to the centers of Ru–M–B2 triangles (but somewhat displaced closer to B2). Populations of these basins are 2.6 *e* in **2-Cu** and 2.0 *e* in **2-Ag**.

The ELF analysis of **2-Au** reveals a substantial change in the bonding situation. The trisynaptic basin observed in **2-Cu** and **2-Ag** is split into two independent disynaptic basins V(Au,B2) and V(Ru,Au). The population value Ω for the V(Au,B2) interaction is 1.9 *e*, which is similar to that of the V(Ru,B1) basin and corresponds to the localized 2c-2e bonding. The V(Ru,Au) basin has the smaller Ω value of 0.7 *e* indicating a weaker 2-center bonding interaction.

Notably, the sum of the V(Ru,Au) and V(Au,B2) basin populations is close to the V(Ru,Cu,B2) basin population in the analogous **2-Cu** complex. The transition of the trisynaptic basin in **2-Cu** and **2-Ag** complexes indicative of 2c-3e bonding to two disynaptic basins in **2-Au** as found in the ELF analysis agrees with the results of the QTAIM analysis discussed above. As there is no direct Ru–B2 bonding, the value of the delocalization index of Ru···B2 interaction can be used as an indicator of the 3-center boding. In **2-Cu** and **2-Ag** this DI value is rather large, 0.31 and 0.33, respectively, whereas in **2-Au** it is reduced to 0.10. In parallel, DI(Ru,M)/DI(M,B2) values are increased from 0.40–0.42/0.66–0.63 in **2-Cu** and **2-Ag** to 0.58/0.81 in **2-Au**. Thus, both QTAIM and ELF analyses show a transition from the less localized three-center B–M···Ru bonding in **2-Cu** and **2-Ag** to the more localized distinct two-center Ru–Au and Au–B2 interactions in **2-Au**.

## Conclusions

The results reported herein uncover unusual reactivity of the ruthenium BB-carboryne with inorganic electrophiles and introduce a new approach for the rational construction of multimetallic complexes supported on the surface of polyhedral boron cages.^11^,^117^ These compounds are the first examples of coordination of electrophilic metal cations to the 2c-2e exohedral metal–boron bonds of boron clusters. The best representation of bonding in **2-Cu** and **2-Ag** complexes is the 3c-2e B–M···Ru interaction description that is often used for bridging B–H···M interactions in boranes and boron clusters while bonding in **2-Au** is closer to the localized 2c-2e Au–B bond. These findings are consistent with the Au–B bond length in **2-Au** being the shortest reported to date. The bonding of Cu and Ag to boron atoms in **2-Cu** and **2-Ag** is also unusually strong with B–M distances comparable to the recently reported isolated 2c-2e bonds of coinage metals with nucleophilic boryls. Importantly, the formation of these complexes represents a unique synthetic strategy for generation of the first examples of the exohedral coinage metal–boryl bonds in {C_2_B_10_} carborane clusters as the direct activation of their B–H bonds by Group 11 metals remains unknown.

Insertion of Cu^+^, Ag^+^, and Au^+^ into strained Ru–B single bonds in **1** is unprecedented. Notably, coordination of coinage metal cations to alkylidenes, silylenes, and borylenes/boryls has been reported.^16^,^19^,^20^,^118^ Recently, coordination of Au^+^ to a platinum–aryl bond has been disclosed.^119^ The results reported herein also open questions whether resembling reactivity can be found in related classes of metal complexes. To the best of our knowledge, analogous metallacycle expansion by coordination of coinage metal cations to metalacyclopropenes or benzyne complexes that are isolobal to **1** has not been reported. It also remains to be seen if similar transformations can be observed in the case of other metal boryls or in the case of other strong inorganic Lewis acids thus providing an alternative synthetic access to novel multimetallic architectures featuring M–B bonds.

## Conflicts of interest

There are no conflicts to declare.

## Supplementary Material

Supplementary information

Supplementary information

Crystal structure data

## References

[cit1] Hirner J. J., Shi Y., Blum S. A. (2011). Acc. Chem. Res..

[cit2] Adams R. D., Captain B. (2009). Acc. Chem. Res..

[cit3] Buchwalter P., Rosé J., Braunstein P. (2015). Chem. Rev..

[cit4] Lohr T. L., Marks T. J. (2015). Nat. Chem..

[cit5] Pye D. R., Mankad N. P. (2017). Chem. Sci..

[cit6] García-Domínguez P., Nevado C. (2016). J. Am. Chem. Soc..

[cit7] Powers I. G., Uyeda C. (2017). ACS Catal..

[cit8] Mankad N. P. (2016). Chem.–Eur. J..

[cit9] Karunananda M. K., Mankad N. P. (2015). J. Am. Chem. Soc..

[cit10] Adams R. D., Captain B., Fu W., Smith M. D. (2002). J. Am. Chem. Soc..

[cit11] Du S., Kautz J. A., McGrath T. D., Stone F. G. A. (2003). Angew. Chem..

[cit12] Chakraborty A., Kinney R. G., Krause J. A., Guan H. (2016). ACS Catal..

[cit13] Tereniak S. J., Carlson R. K., Clouston L. J., Young V. G., Bill E., Maurice R., Chen Y.-S., Kim H. J., Gagliardi L., Lu C. C. (2014). J. Am. Chem. Soc..

[cit14] Uyeda C., Peters J. C. (2013). J. Am. Chem. Soc..

[cit15] Cammarota R. C., Lu C. C. (2015). J. Am. Chem. Soc..

[cit16] Stone F. G. A. (1984). Angew. Chem., Int. Ed..

[cit17] Dossett S. J., Hill A. F., Jeffery J. C., Marken F., Sherwood P., Stone F. G. A. (1988). J. Chem. Soc., Dalton Trans..

[cit18] Raubenheimer H. G., Schmidbaur H. (2012). Organometallics.

[cit19] Liu H.-J., Raynaud C., Eisenstein O., Tilley T. D. (2014). J. Am. Chem. Soc..

[cit20] Braunschweig H., Radacki K., Shang R. (2015). Chem. Sci..

[cit21] Irvine G. J., Lesley M. J. G., Marder T. B., Norman N. C., Rice C. R., Robins E. G., Roper W. R., Whittell G. R., Wright L. J. (1998). Chem. Rev..

[cit22] Braunschweig H., Kollann C., Rais D. (2006). Angew. Chem., Int. Ed..

[cit23] Braunschweig H., Dewhurst R. D., Schneider A. (2010). Chem. Rev..

[cit24] Dang L., Lin Z., Marder T. B. (2009). Chem. Commun..

[cit25] Segawa Y., Yamashita M., Nozaki K. (2006). Science.

[cit26] Press L. P., Kosanovich A. J., McCulloch B. J., Ozerov O. V. (2016). J. Am. Chem. Soc..

[cit27] Shih W.-C., Gu W., MacInnis M. C., Timpa S. D., Bhuvanesh N., Zhou J., Ozerov O. V. (2016). J. Am. Chem. Soc..

[cit28] Shih W.-C., Ozerov O. V. (2017). J. Am. Chem. Soc..

[cit29] Grimes R. N. (2015). Dalton Trans..

[cit30] Yamashita M. (2016). Bull. Chem. Soc. Jpn..

[cit31] Hoel E. L., Hawthorne M. F. (1975). J. Am. Chem. Soc..

[cit32] Saleh L. M. A., Dziedzic R. M., Khan S. I., Spokoyny A. M. (2016). Chem.–Eur. J..

[cit33] Li Y., Sneddon L. G. (2008). J. Am. Chem. Soc..

[cit34] Asay M., Kefalidis C. E., Estrada J., Weinberger D. S., Wright J., Moore C. E., Rheingold A. L., Maron L., Lavallo V. (2013). Angew. Chem., Int. Ed..

[cit35] Spokoyny A. M. (2013). Pure Appl. Chem..

[cit36] Poater J., Solà M., Viñas C., Teixidor F. (2014). Angew. Chem., Int. Ed..

[cit37] Selg C., Neumann W., Lönnecke P., Hey-Hawkins E., Zeitler K. (2017). Chem.–Eur. J..

[cit38] Popescu A. R., Teixidor F., Viñas C. (2014). Coord. Chem. Rev..

[cit39] Juhasz M., Hoffmann S., Stoyanov E., Kim K.-C., Reed C. A. (2004). Angew. Chem., Int. Ed..

[cit40] Dash B. P., Satapathy R., Gaillard E. R., Maguire J. A., Hosmane N. S. (2010). J. Am. Chem. Soc..

[cit41] Douvris C., Ozerov O. V. (2008). Science.

[cit42] Xie Z. (2002). Acc. Chem. Res..

[cit43] El-Hellani A., Lavallo V. (2014). Angew. Chem., Int. Ed..

[cit44] Chan A. L., Estrada J., Kefalidis C. E., Lavallo V. (2016). Organometallics.

[cit45] McArthur S. G., Jay R., Geng L., Guo J., Lavallo V. (2017). Chem. Commun..

[cit46] Rodríguez-Hermida S., Tsang M. Y., Vignatti C., Stylianou K. C., Guillerm V., Pérez-Carvajal J., Teixidor F., Viñas C., Choquesillo-Lazarte D., Verdugo-Escamilla C., Peral I., Juanhuix J., Verdaguer A., Imaz I., Maspoch D., Giner Planas J. (2016). Angew. Chem., Int. Ed..

[cit47] Joost M., Estévez L., Miqueu K., Amgoune A., Bourissou D. (2015). Angew. Chem., Int. Ed..

[cit48] Núñez R., Romero I., Teixidor F., Viñas C. (2016). Chem. Soc. Rev..

[cit49] Axtell J. C., Kirlikovali K. O., Djurovich P. I., Jung D., Nguyen V. T., Munekiyo B., Royappa A. T., Rheingold A. L., Spokoyny A. M. (2016). J. Am. Chem. Soc..

[cit50] Messina M. S., Axtell J. C., Wang Y., Chong P., Wixtrom A. I., Kirlikovali K. O., Upton B. M., Hunter B. M., Shafaat O. S., Khan S. I., Winkler J. R., Gray H. B., Alexandrova A. N., Maynard H. D., Spokoyny A. M. (2016). J. Am. Chem. Soc..

[cit51] Kwan E. H., Ogawa H., Yamashita M. (2017). ChemCatChem.

[cit52] Wang H., Zhang J., Xie Z. (2017). Angew. Chem., Int. Ed..

[cit53] Estrada J., Lavallo V. (2017). Angew. Chem., Int. Ed..

[cit54] Sayler A. A., Beall H., Sieckhaus J. F. (1973). J. Am. Chem. Soc..

[cit55] Wang H., Li H.-W., Huang X., Lin Z., Xie Z. (2003). Angew. Chem., Int. Ed..

[cit56] Zhao D., Zhang J., Xie Z. (2014). Angew. Chem., Int. Ed..

[cit57] Qiu Z., Ren S., Xie Z. (2011). Acc. Chem. Res..

[cit58] Qiu Z., Xie Z. (2014). Dalton Trans..

[cit59] Eleazer B. J., Smith M. D., Popov A. A., Peryshkov D. V. (2016). J. Am. Chem. Soc..

[cit60] Laitar D. S., Müller P., Sadighi J. P. (2005). J. Am. Chem. Soc..

[cit61] Segawa Y., Yamashita M., Nozaki K. (2007). Angew. Chem., Int. Ed..

[cit62] Braunschweig H., Damme A., Dewhurst R. D., Kramer T., Östreicher S., Radacki K., Vargas A. (2013). J. Am. Chem. Soc..

[cit63] Borner C., Kleeberg C. (2014). Eur. J. Inorg. Chem..

[cit64] Kajiwara T., Terabayashi T., Yamashita M., Nozaki K. (2008). Angew. Chem., Int. Ed..

[cit65] Semba K., Shinomiya M., Fujihara T., Terao J., Tsuji Y. (2013). Chem.–Eur. J..

[cit66] Braunschweig H., Ewing W. C., Kramer T., Mattock J. D., Vargas A., Werner C. (2015). Chem.–Eur. J..

[cit67] Hodson B. E., McGrath T. D., Stone F. G. A. (2004). Dalton Trans..

[cit68] Ellis D. D., Couchman S. M., Jeffery J. C., Malget J. M., Stone F. G. A. (1999). Inorg. Chem..

[cit69] Ellis D. D., Franken A., Stone F. G. A. (1999). Organometallics.

[cit70] Do Y., Kang H. C., Knobler C. B., Hawthorne M. F. (1987). Inorg. Chem..

[cit71] Du S., Kautz J. A., McGrath T. D., Stone F. G. A. (2003). Dalton Trans..

[cit72] Hodson B. E., McGrath T. D., Stone F. G. A. (2005). Organometallics.

[cit73] Ellis D. D., Jelliss P. A., Stone F. G. A. (1999). Organometallics.

[cit74] Hodson B. E., McGrath T. D., Stone F. G. A. (2005). Organometallics.

[cit75] Batten S. A., Jeffery J. C., Jones P. L., Mullica D. F., Rudd M. D., Sappenfield E. L., Stone F. G. A., Wolf A. (1997). Inorg. Chem..

[cit76] Hata M., Kautz J. A., Lu X. L., McGrath T. D., Stone F. G. A. (2004). Organometallics.

[cit77] Banerjee S., Karunananda M. K., Bagherzadeh S., Jayarathne U., Parmelee S. R., Waldhart G. W., Mankad N. P. (2014). Inorg. Chem..

[cit78] Brown S. S. D., Salter I. D., Toupet L. (1988). J. Chem. Soc., Dalton Trans..

[cit79] Draper S. M., Hattersley A. D., Housecroft C. E., Rheingold A. L. (1992). J. Chem. Soc., Chem. Commun..

[cit80] Brown S. S. D., Hudson S., Salter I. D., McPartlin M. (1987). J. Chem. Soc., Dalton Trans..

[cit81] Nakajima T., Konomoto H., Ogawa H., Wakatsuki Y. (2007). J. Organomet. Chem..

[cit82] Shieh M., Miu C.-Y., Hsing K.-J., Jang L.-F., Lin C.-N. (2015). Dalton Trans..

[cit83] Bradley J. S., Pruett R. L., Hill E., Ansell G. B., Leonowicz M. E., Modrick M. A. (1982). Organometallics.

[cit84] Deng H., Shore S. G. (1991). Organometallics.

[cit85] Brown C. J., McCarthy P. J., Salter I. D., Armstrong K. P., McPartlin M., Powell H. R. (1990). J. Organomet. Chem..

[cit86] Evans J., Stroud P. M., Webster M. (1989). Organometallics.

[cit87] Beswick M. A., Lewis J., Raithby P. R., de Arellano M. C. R. (1997). Angew. Chem., Int. Ed. Engl..

[cit88] Riley L. E., Chan A. P. Y., Taylor J., Man W. Y., Ellis D., Rosair G. M., Welch A. J., Sivaev I. B. (2016). Dalton Trans..

[cit89] Liu D., Dang L., Sun Y., Chan H.-S., Lin Z., Xie Z. (2008). J. Am. Chem. Soc..

[cit90] Herberhold M., Yan H., Milius W., Wrackmeyer B. (2002). Chem.–Eur. J..

[cit91] Viñas C., Nuñez R., Teixidor F., Kivekäs R., Sillanpää R. (1996). Organometallics.

[cit92] Teixidor F., Vinas C., Casabo J., Romerosa A. M., Rius J., Miravitlles C. (1994). Organometallics.

[cit93] Teixidor F., Ayllon J. A., Vinas C., Kivekas R., Sillanpaa R., Casabo J. (1994). Organometallics.

[cit94] Teixidor F., Flores M. A., Viñas C., Kivekäs R., Sillanpää R. (1998). Organometallics.

[cit95] Ellis D. D., Franken A., Jelliss P. A., Kautz J. A., Stone F. G. A., Yu P.-Y. (2000). J. Chem. Soc., Dalton Trans..

[cit96] Eleazer B. J., Smith M. D., Peryshkov D. V. (2016). Organometallics.

[cit97] Dahl E. W., Szymczak N. K. (2016). Angew. Chem., Int. Ed..

[cit98] Gagne R. R., Allison J. L., Lisensky G. C. (1978). Inorg. Chem..

[cit99] Balogh-Hergovich É., Kaizer J., Speier G., Huttner G., Jacobi A. (2000). Inorg. Chem..

[cit100] Shinoura M., Kita S., Ohba M., Ōkawa H., Furutachi H., Suzuki M. (2000). Inorg. Chem..

[cit101] Arnold P. L., Scarisbrick A. C., Blake A. J., Wilson C. (2001). Chem. Commun..

[cit102] Lu W., Hu H., Li Y., Ganguly R., Kinjo R. (2016). J. Am. Chem. Soc..

[cit103] Alexander B. D., Johnson B. J., Johnson S. M., Boyle P. D., Kann N. C., Mueting A. M., Pignolet L. H. (1987). Inorg. Chem..

[cit104] Wade C. R., Gabbaï F. P. (2011). Angew. Chem., Int. Ed..

[cit105] Yang H., Gabbaï F. P. (2015). J. Am. Chem. Soc..

[cit106] Sen S., Ke I.-S., Gabbaï F. P. (2016). Inorg. Chem..

[cit107] Braunschweig H., Brunecker C., Dewhurst R. D., Schneider C., Wennemann B. (2015). Chem.–Eur. J..

[cit108] Bruce M. I., Williams M. L., Patrick J. M., Skelton B. W., White A. H. (1986). J. Chem. Soc., Dalton Trans..

[cit109] Bader R. F. W., Atoms in Molecules: A Quantum Theory, Oxford University Press, Oxford, New York, 1994.

[cit110] Bader R. F. W., Legare D. A. (1992). Can. J. Chem..

[cit111] Keith T. A., AIMAll, 2014.

[cit112] Multiwfn code: Lu T. Chen F., J. Comput. Chem., 2012, 33 , 580 –592 . 22162017 10.1002/jcc.22885

[cit113] Savin A., Nesper R., Wengert S., Fässler T. F. (1997). Angew. Chem., Int. Ed. Engl..

[cit114] TopMoD code: Noury S. Krokidis X. Fuster F. Silvi B., Comput. Chem., 1999, 23 , 597 –604 .

[cit115] Neese F. (2012). Wiley Interdiscip. Rev.: Comput. Mol. Sci..

[cit116] Pantazis D. A., Chen X.-Y., Landis C. R., Neese F. (2008). J. Chem. Theory Comput..

[cit117] Adams R. D., Kiprotich J., Peryshkov D. V., Wong Y. O. (2016). Chem.–Eur. J..

[cit118] Braunschweig H., Radackia K., Shanga R. (2013). Chem. Commun..

[cit119] Baya M., Belío Ú., Fernández I., Fuertes S., Martín A. (2016). Angew. Chem., Int. Ed..

