# Supplementary material for: Expansion of the (BB) 〉metallacycle with coinage metal cations: formation of B–M–Ru–B (M = Cu, Ag, Au) dimetalacyclodiboryls
Source: Chem Sci. 2018 Feb 5;9(9):2601–8. doi: 10.1039/c8sc00190a (PMC5892348; doi:10.1039/c8sc00190a)
Supplement: Supplementary file 2 [file SC-009-C8SC00190A-s002.pdf]

Electronic Supplementary Material (ESI) for Chemical Science.
This journal is © The Royal Society of Chemistry 2018

Electronic Supplementary Material (ESI)

Expansion of the (BB)>Ru Metallacycle with Coinage Metal Cations: Formation of B-M-Ru-B (M = Cu, Ag, Au) Dimetalacyclodiboryls

Bennett J. Eleazer[a], Mark D. Smith[a], Alexey A. Popov[b]*, and Dmitry V. Peryshkov[a]*.

a). Department of Chemistry and Biochemistry, University of South Carolina, 631 Sumter St., Columbia, South Carolina 29208, United States.
b). Leibniz Institute for Solid State and Materials Research, Helmholtzstrasse 20, 01069 Dresden, Germany.

Experimental

All synthetic manipulations were carried out either in a nitrogen-filled drybox or on an air-free dual-manifold Schlenk line, unless stated otherwise. Compounds **2-Cu**, **2-Au**, and **2-Ag** were found to be moderately stable on air in solid state. Solvents were sparged with nitrogen, passed through activated alumina, and stored over activated 4 Å Linde-type molecular sieves. Benzene-d_6 and dichloromethane-d_2 were degassed and stored over activated 4 Å Linde-type molecular sieves. NMR spectra were recorded using Varian spectrometers at 400 (^{1}H), 100 (^{13}C), 162 (^{31}P), 128 (^{11}B) MHz, reported in δ (parts per million) and referenced to the residual ^{1}H/^{13}C signals of the deuterated solvent or an external 85% H_3PO_4 (^{31}P (δ): 0.0 ppm) and $BF_3(Et_2O)$ (^{11}B(δ): 0.0 ppm) standards. J values are given in Hz. Midwest Microlab, Indianapolis, Indiana provided the elemental analysis results.

(POBBOP)Ru(CO)$_2$ carboryne (POBBOP = 1,7-OP(*i*-Pr)$_2$-*m*-2,6-dehydrocarborane) was prepared using the previously reported procedure.[1] *m*-carborane $C_2B_{10}H_{12}$ (Katchem) was used as received.

Synthesis of [(POBBOP)(Ru)(CO)$_2$(Cu)(Cl)]$_2$ complex (2-Cu)

A portion of CuCl (0.009 g; 0.091 mmol) was added to a solution of (POBBOP)Ru(CO)$_2$ (0.050g; 0.089 mmol) in C_6D_6 (0.5 mL) and tetrahydrofuran (1 mL). The reaction mixture was then transferred to a J Young valve NMR tube and heated for 12 hours at 90 °C. The resulting solution was dried under vacuum, triturated with hexanes (3 × 1 mL) and acetonitrile (1 mL), and filtered. The tetrahydrofuran extract was evaporated producing a white solid. Yield: 0.041 g, 0.031 mmol, 70%.

^{1}H NMR (C_6D_6): δ 2.26 (br, 2H, (PC*H*(CH$_3$)$_2$), 1.91 (br, 2H, (PC*H*(CH$_3$)$_2$), 1.29 (m, 6H, (PCH(C*H*$_3$)$_2$), 0.87 (m, 6H, (PCH(C*H*$_3$)$_2$), 0.76 (m, 6H, (PCH(C*H*$_3$)$_2$), 0.66 (m, 6H, (PCH(C*H*$_3$)$_2$). **^{11}B{^{1}H}** (C_6D_6): δ -1.5 (B-Ru), -10.3 (B-Cu), -11.0 (B-H), -12.2 (B-H), -12.9 (B-H), -15.5 (B-H). **^{13}C (**CDCl$_3$**):** δ 111.9 (*C*$_2$B$_{10}$H$_{10}$), 33.7 (P*C*H(CH$_3$)$_2$), 18.1 (PCH(*C*H$_3$)$_2$), 16.8 (PCH(*C*H$_3$)$_2$). **^{31}P{^{1}H}** (C_6D_6): δ 204.2. Selected bands in the IR spectrum, cm^{-1}: 2990–2880 (br, CH), 2600 (br, BH), 2020 (strong, Ru-*CO*), 1969 (strong, Ru-*CO*). Found: C, 29.29; H, 5.53. Calcd. for $C_{32}H_{72}B_{20}Cl_2Cu_2O_8P_4Ru_2$: C, 29.00; H, 5.48.

Synthesis of [(POBBOP)(Ru)(CO)$_2$(Au)(Cl)]$_2$ complex (2-Au)

A solution of Au(SMe$_2$)Cl (0.026 g; 0.088 mmol) in CH_2Cl_2 (3 mL) was added to a solution of (POBBOP)Ru(CO)$_2$ (0.050 g; 0.088 mmol) in CH_2Cl_2 (3 mL). The reaction mixture was stirred at room temperature overnight. The resulting solution was dried under vacuum, triturated with hexanes (3 ×1 mL) and acetonitrile (1mL), and filtered. The CH_2Cl_2 extract was evaporated producing a white solid. Yield: 0.061 g, 0.036 mmol, 81%.

^{1}H NMR (CD_2Cl_2): δ 2.51 (m, 4H, (PC*H*(CH_3)$_2$), 1.30 (m, 12H, (PCH(C*H*$_3$)$_2$), 1.21 (m, 6H, (PCH(C*H*$_3$)$_2$), 1.07 (m, 6H, (PCH(C*H*$_3$)$_2$). **^{11}B{^{1}H}** (CD_2Cl_2): δ 2.1 (*B*-Ru), -9.7 (*B*-Au), -11.7 (*B*-H), -13.1 (*B*-H), -15.6 (*B*-H), -16.8 (*B*-H). **^{13}C (**CD_2Cl_2**):** δ 109.0 (*C*$_2$B$_{10}$H$_{10}$), 36.3 (P*C*H(CH_3)$_2$), 28.6 (P*C*H(CH_3)$_2$), 19.8 (PCH(*C*H$_3$)$_2$), 17.7 (PCH(*C*H$_3$)$_2$), 15.7 (PCH(*C*H$_3$)$_2$), 15.2 (PCH(*C*H$_3$)$_2$). **^{31}P{^{1}H}** (CD_2Cl_2): δ 205.1. Selected bands in the IR spectrum, cm^{-1}: 2992–2878 (br, CH), 2590 (br, BH), 2038 (strong, Ru-*CO*), 1966 (strong, Ru-*CO*). Found: C, 24.38; H, 4.19. Calcd. for $C_{32}H_{72}Au_2B_{20}Cl_2O_8P_4Ru_2$: C, 24.14; H, 4.56.

Synthesis of (POBBOP)(Ru)(CO)$_2$(Ag)(CH_3CN)(NO_3) complex (2-Ag)

A portion of $AgNO_3$ (0.015 g; 0.088 mmol) was added to a solution of (POBBOP)Ru(CO)$_2$ (0.050 g; 0.089 mmol) in C_6D_6 (1 mL) and CH_2Cl_2 (1 mL). The reaction mixture was then transferred to a J Young valve NMR tube and heated for 12 hours at 50 °C. The resulting solution was dried under vacuum, triturated with hexanes (3 × 1 mL) and acetonitrile (1mL), and filtered. The CH_2Cl_2 extract was evaporated producing a white solid. Yield: 0.045 g, 0.061 mmol, 70%.

^{1}H NMR (C_6D_6): δ 1.97 (br, 2H, (PC*H*(CH_3)$_2$), 1.85 (br, 2H, (PC*H*(CH_3)$_2$), 1.04 (m, 6H, (PCH(C*H*$_3$)$_2$), 0.78 (m, 12H, (PCH(C*H*$_3$)$_2$), 0.65 (m, 6H, (PCH(C*H*$_3$)$_2$). **^{11}B{^{1}H}** (C_6D_6): δ -2.7 (*B*-Ru), -11.9 (*B*-Ag), -12.8 (*B*-H), -15.0 (*B*-H), -15.4 (*B*-H). **^{13}C (**C_6D_6**):** δ 199.5 (Ru-*C*O), 196.8 (Ru-*C*O), 117.5 (CH$_3$*C*N) 111.4 (*C*$_2$B$_{10}$H$_{10}$), 35.2 (P*C*H(CH_3)$_2$), 30.5 (P*C*H(CH_3)$_2$), 17.8 (PCH(*C*H$_3$)$_2$), 15.5 (PCH(*C*H$_3$)$_2$), 0.7 (*C*H$_3$CN). **^{31}P{^{1}H}** (C_6D_6): δ 205.5. Selected bands in the IR spectrum, cm^{-1}: 2971–2882 (br, CH), 2594 (br, BH), 2283 (br, Ru-*N*CCH$_3$), 2038 (strong, Ru-*CO*), 1995 (strong, Ru-*CO*).

Found: C, 28.35; H, 4.84; N, 3.21. Calcd. for $C_{18}H_{39}AgB_{10}N_2O_7P_2Ru$: C, 27.91; H, 5.08; N, 3.62.

References:

1. Eleazer, B. J.; Smith, M. D.; Popov, A. A.; Peryshkov, D. V. *J. Am. Chem. Soc.*, **2016**, *138*, 10531–10538.

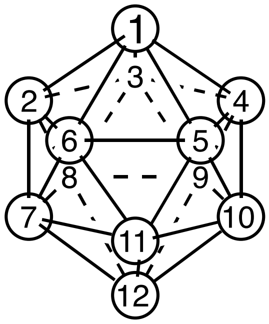

Figure S-1. The numbering scheme for the icosahedral *closo*-carborane cluster. For the *m*-$C_2B_{10}H_{12}$, carbon atoms are at positions 1 and 7.

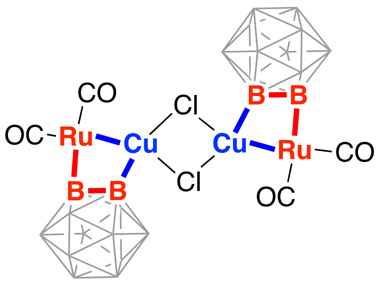

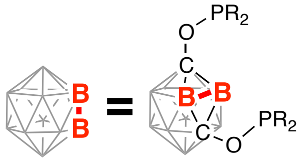

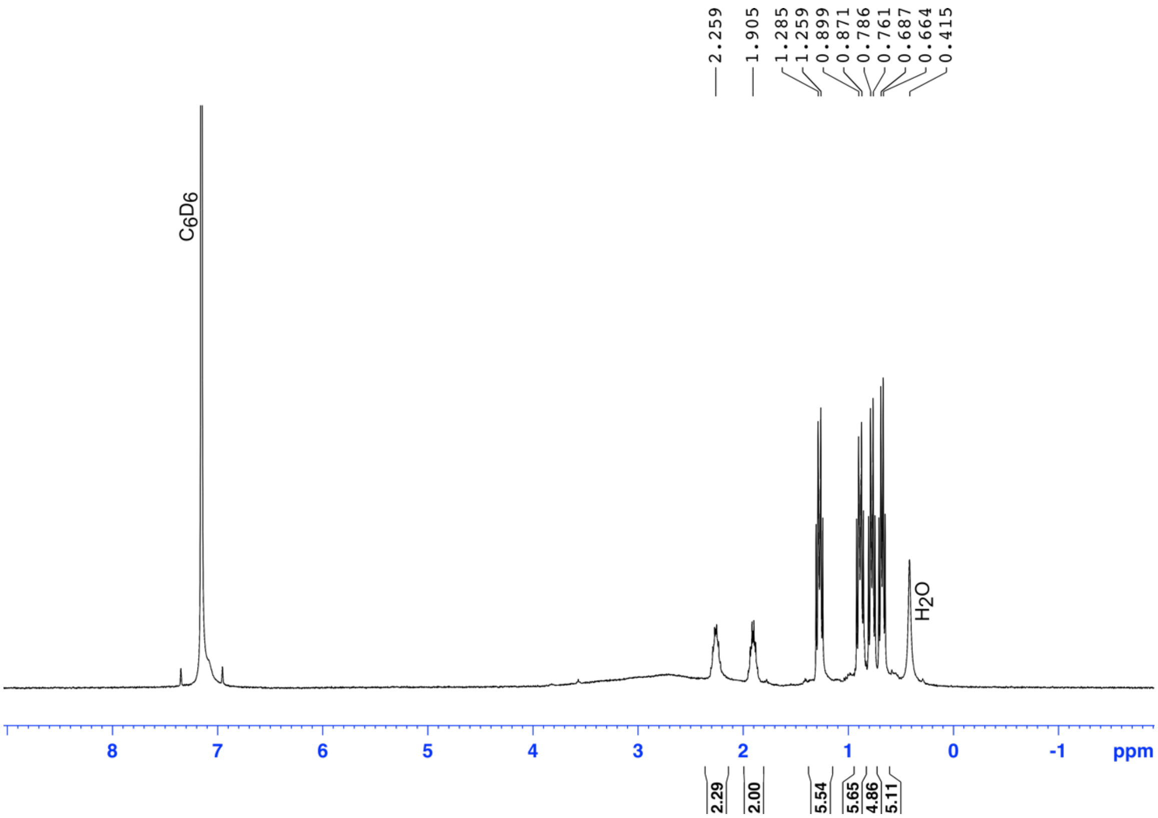

Figure S-2. The ^{1}H NMR spectrum of [(POBBOP)(Ru)(CO)$_2$(Cu)(Cl)]$_2$ (**2-Cu**) in C_6D_6.

^{1}H NMR (C_6D_6): δ 2.26 (br, 2H, (PC*H*(CH$_3$)$_2$), 1.91 (br, 2H, (PC*H*(CH$_3$)$_2$), 1.29 (m, 6H, (PCH(C*H*$_3$)$_2$), 0.87 (m, 6H, (PCH(C*H*$_3$)$_2$), 0.76 (m, 6H, (PCH(C*H*$_3$)$_2$), 0.66 (m, 6H, (PCH(C*H*$_3$)$_2$).

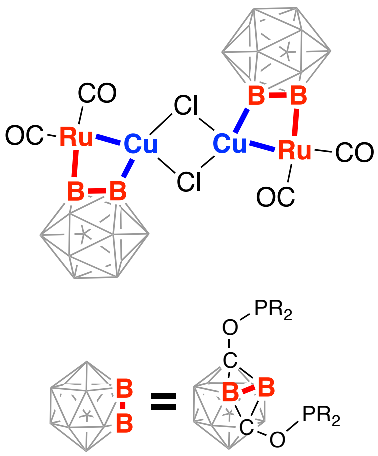

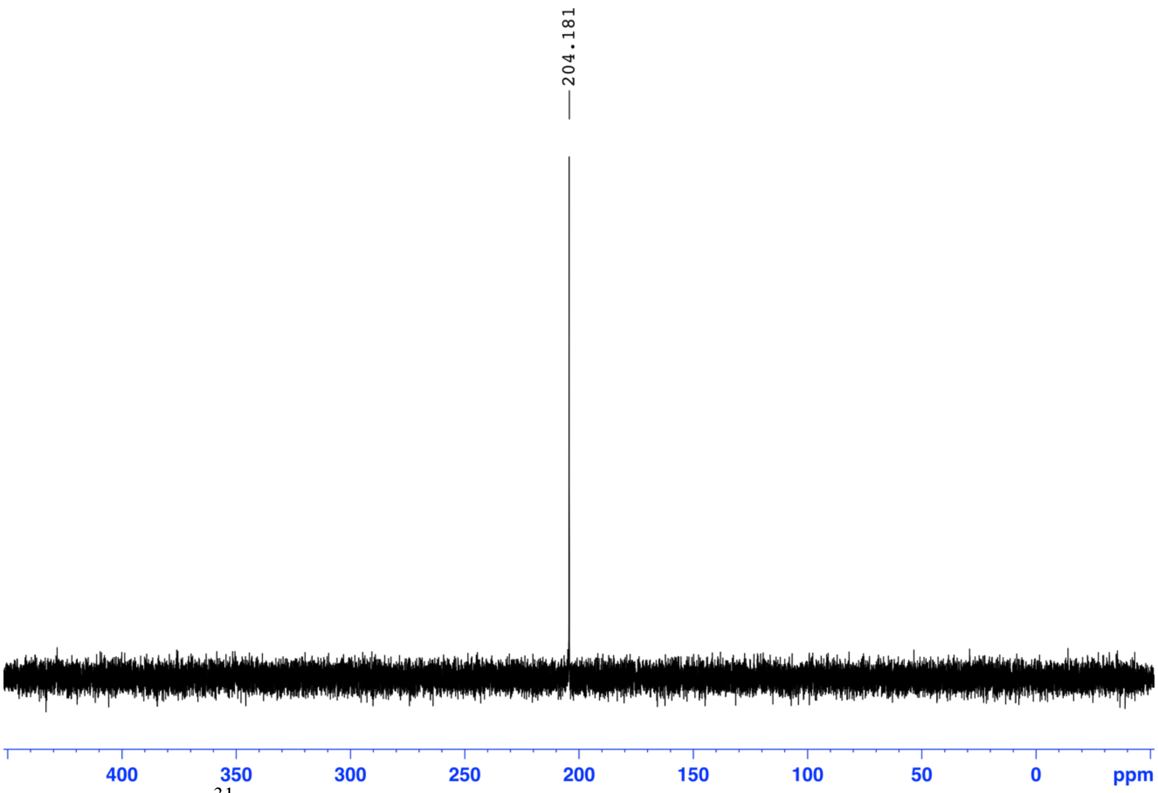

Figure S-3. The ^{31}P NMR spectrum of [(POBBOP)(Ru)(CO)$_2$(Cu)(Cl)]$_2$ (**2-Cu**) in C_6D_6.

^{31}P{^{1}H} (C_6D_6): δ 204.2.

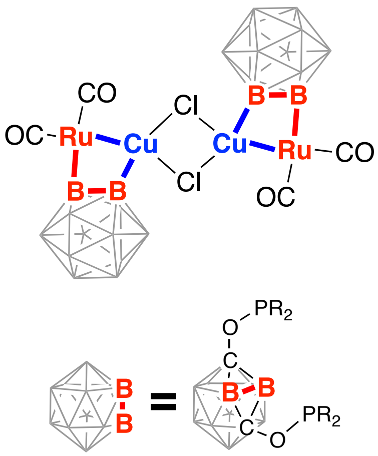

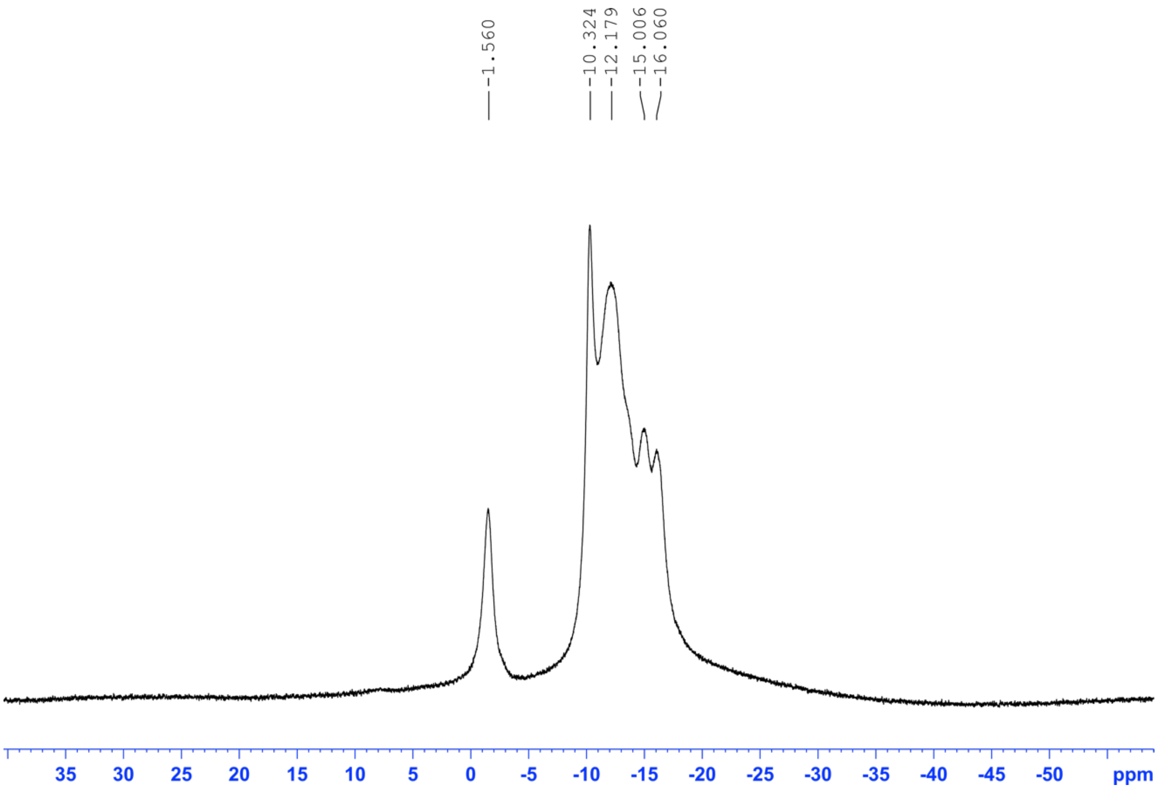

Figure S-4. The ^{11}B NMR spectrum of [(POBBOP)(Ru)$(CO)_2$(Cu)(Cl)]$_2$ (**2-Cu**) in C_6D_6.

$^{11}B\{^1H\}$ (C_6D_6): δ -1.5 (B-Ru), -10.3 (B-Cu), -11.0 (B-H), -12.2 (B-H), -12.9 (B-H), -15.5 (B-H).

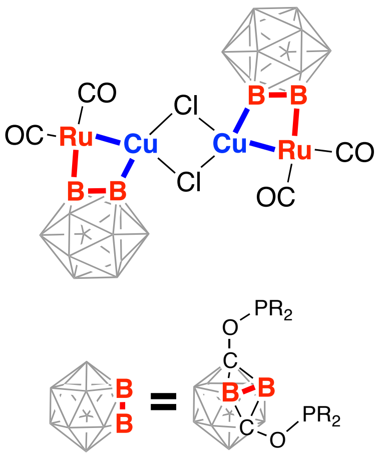

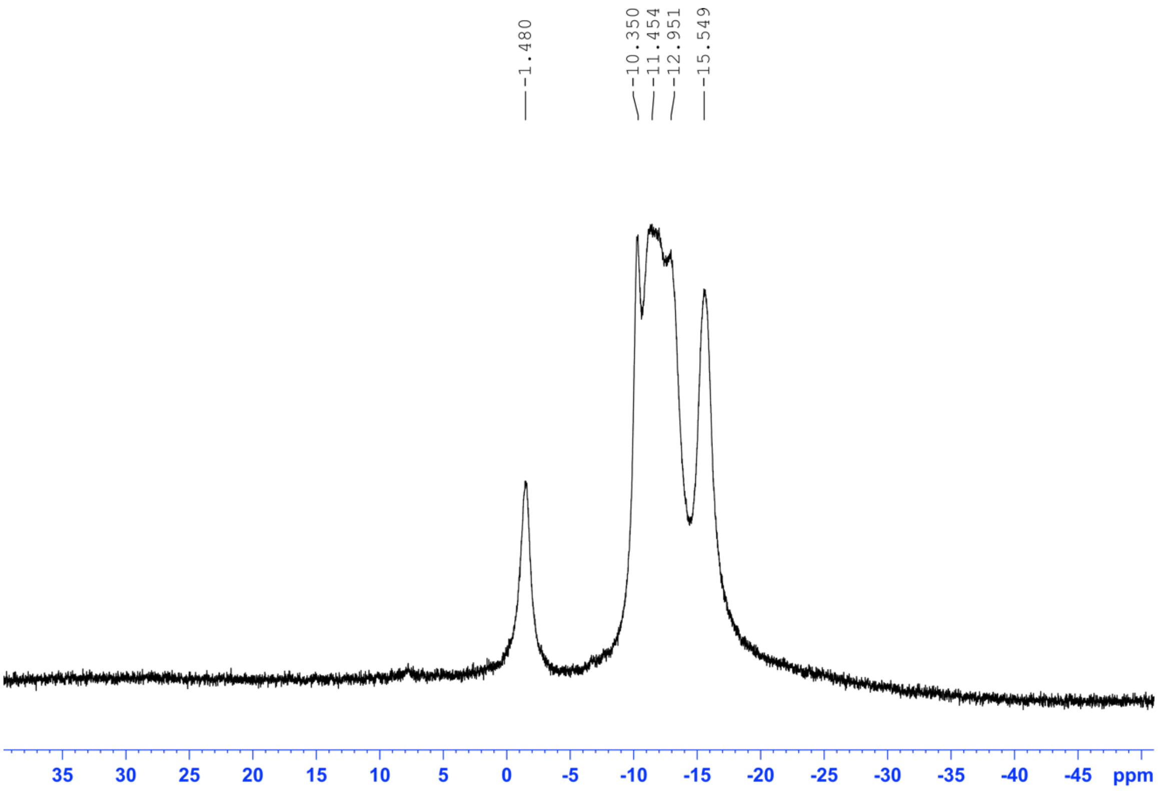

Figure S-5. The $^{11}B\{^1H\}$ NMR spectrum of [(POBBOP)(Ru)(CO)$_2$(Cu)(Cl)]$_2$ (**2-Cu**) in C_6D_6.

^{11}B{^{1}H} (C_6D_6): δ -1.5 (B-Ru), -10.3 (B-Cu), -11.0 (B-H), -12.2 (B-H), -12.9 (B-H), -15.5 (B-H).

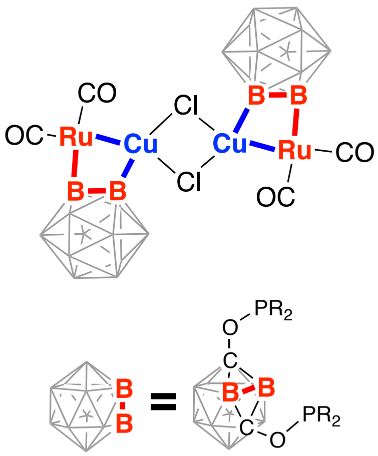

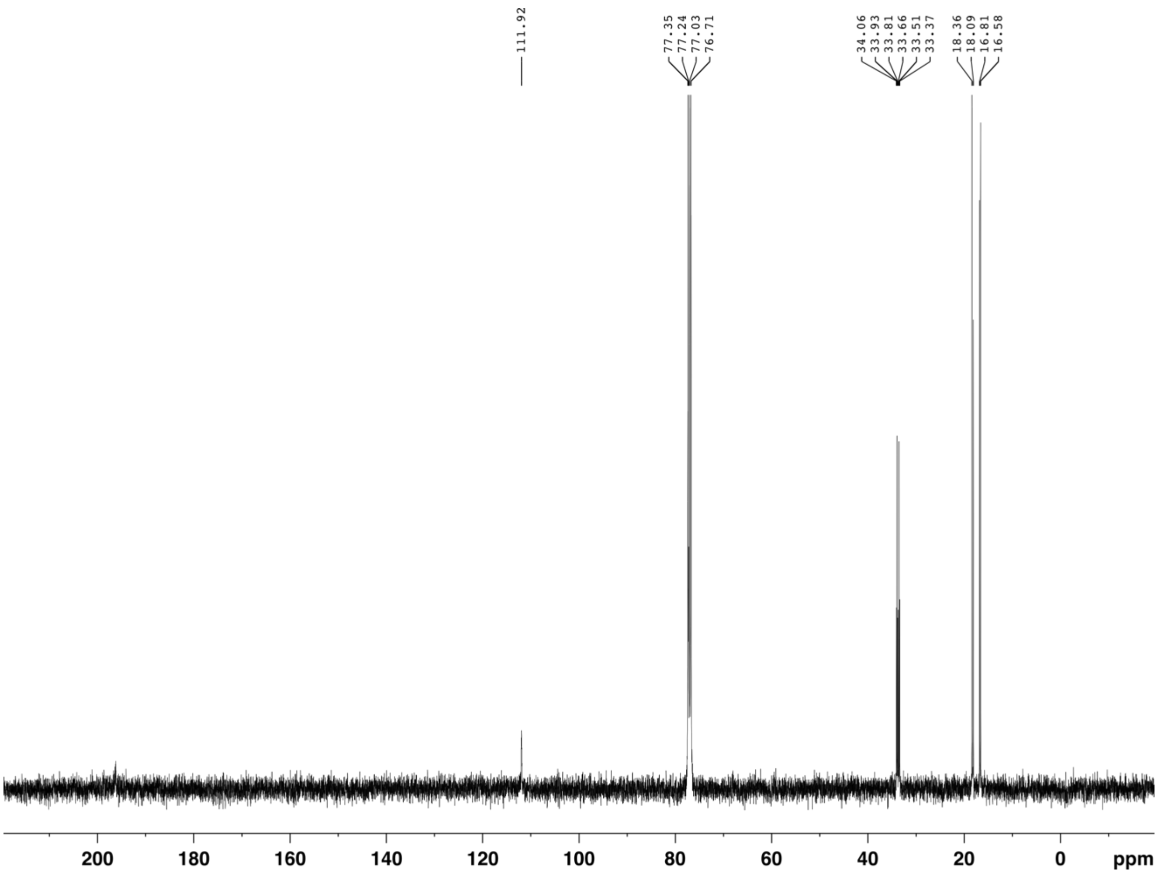

Figure S-6. The ^{13}C NMR spectrum of $[(POBBOP)(Ru)(CO)_2(Cu)(Cl)]_2$ (**2-Cu**) in C_6D_6.

^{13}C ($CDCl_3$**):** δ 111.9 ($C_2B_{10}H_{10}$), 33.7 (P*C*H(CH)$_3$)$_2$), 18.1 (PCH(*C*H)$_3$)$_2$), 16.8 (PCH(*C*H)$_3$)$_2$).

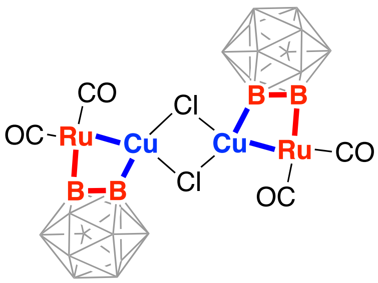

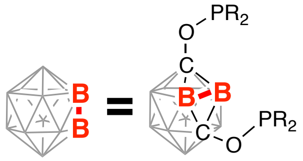

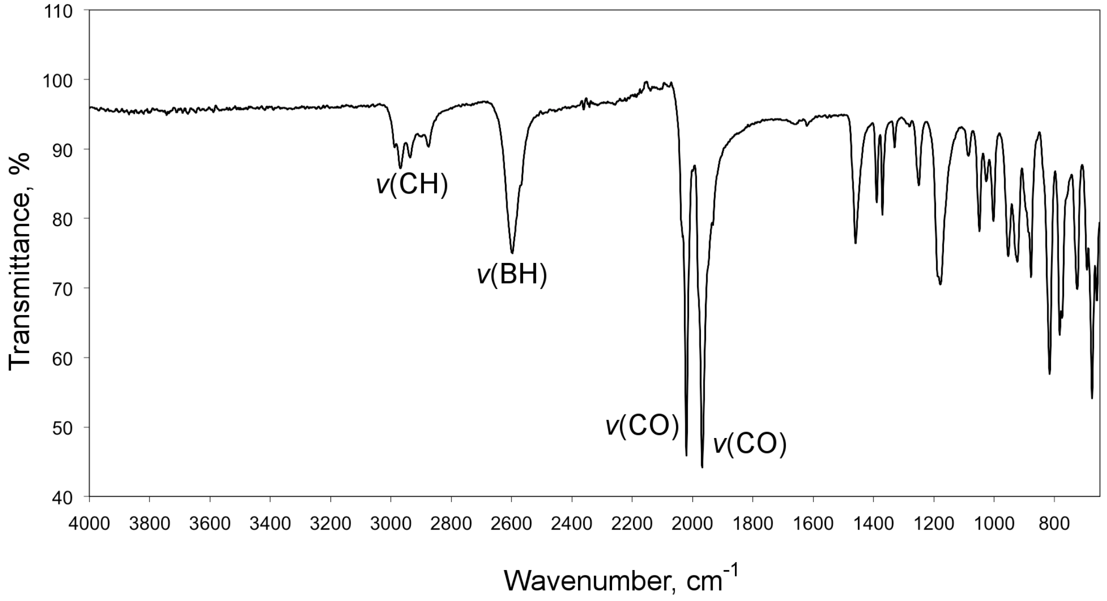

Figure S-7. The ATR-FTIR absorption spectrum of a solid sample of $[(POBBOP)(Ru)(CO)_2(Cu)(Cl)]_2$ (**2-Cu**).

Selected bands in the IR spectrum, cm^{-1}: 2990–2880 (br, CH), 2600 (br, BH), 2020 (strong, Ru-*CO*), 1969 (strong, Ru-*CO*).

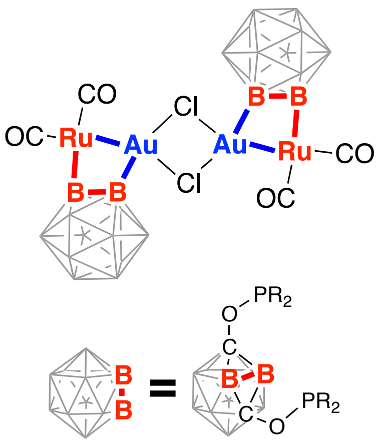

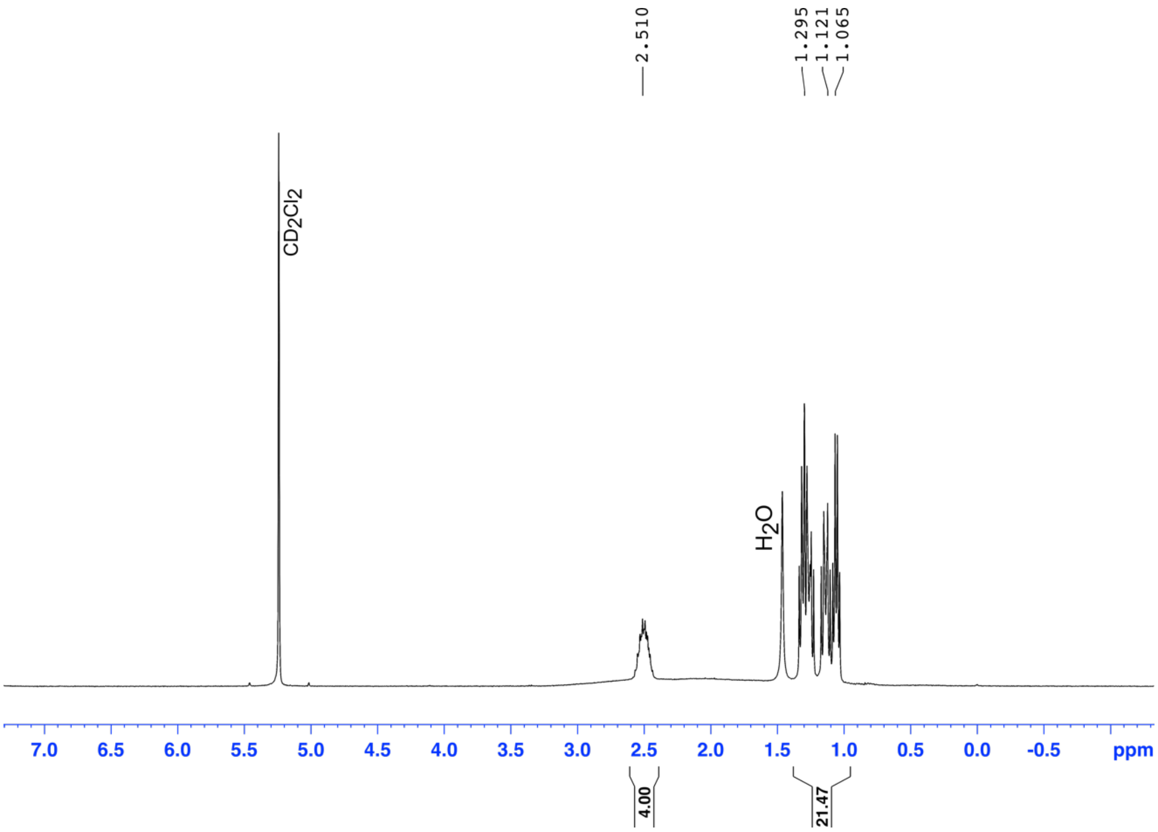

Figure S-8. The ^{1}H NMR spectrum of $[(POBBOP)(Ru)(CO)_2(Au)(Cl)]_2$ (**2-Au**) in CD_2Cl_2.

^{1}H NMR (CD_2Cl_2): δ 2.51 (m, 4H, (PC*H*(CH_3)$_2$), 1.30 (m, 12H, (PCH(C*H*$_3$)$_2$), 1.21 (m, 6H, (PCH(C*H*$_3$)$_2$), 1.07 (m, 6H, (PCH(C*H*$_3$)$_2$).

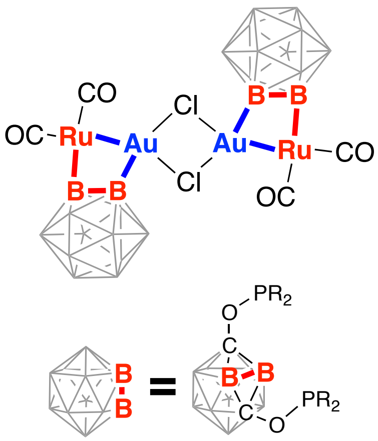

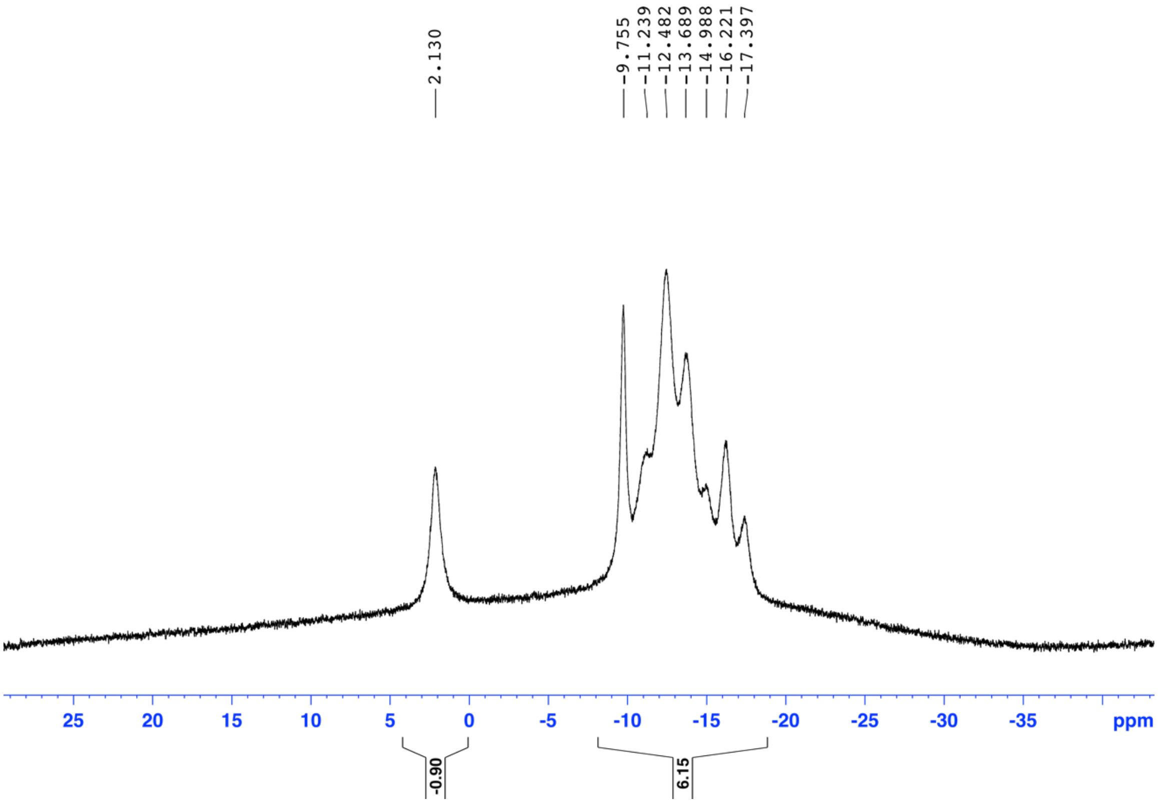

Figure S-9. The ^{11}B NMR spectrum of $[(POBBOP)(Ru)(CO)_2(Au)(Cl)]_2$ (**2-Au**) in CD_2Cl_2.

^{11}B{^{1}H} (CD_2Cl_2): δ 2.1 (*B*-Ru), -9.7 (*B*-Au), -11.7 (*B*-H), -13.1 (*B*-H), -15.6 (*B*-H), -16.8 (*B*-H).

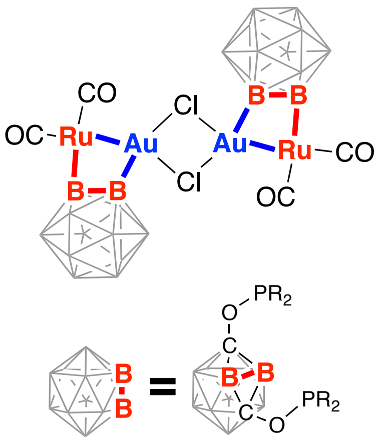

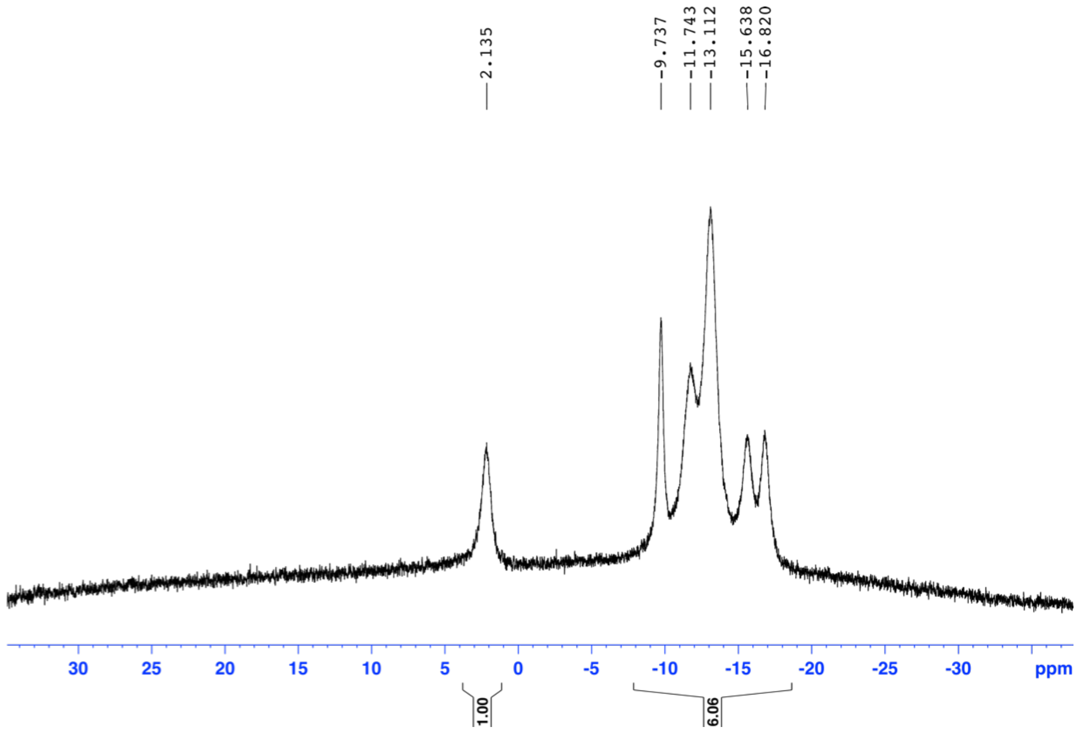

Figure S-10. The $^{11}B\{^1H\}$ NMR spectrum of [(POBBOP)(Ru)(CO)$_2$(Au)(Cl)]$_2$ (**2-Au**) in CD_2Cl_2.

$^{11}B\{^1H\}$ (CD_2Cl_2): δ 2.1 (*B*-Ru), -9.7 (*B*-Au), -11.7 (*B*-H), -13.1 (*B*-H), -15.6 (*B*-H), -16.8 (*B*-H).

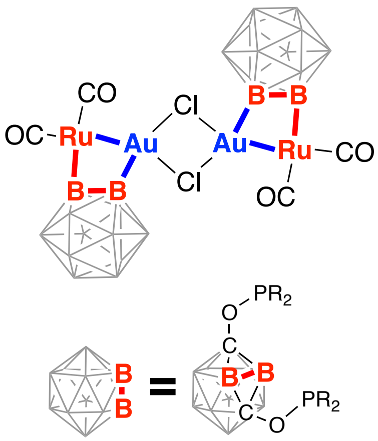

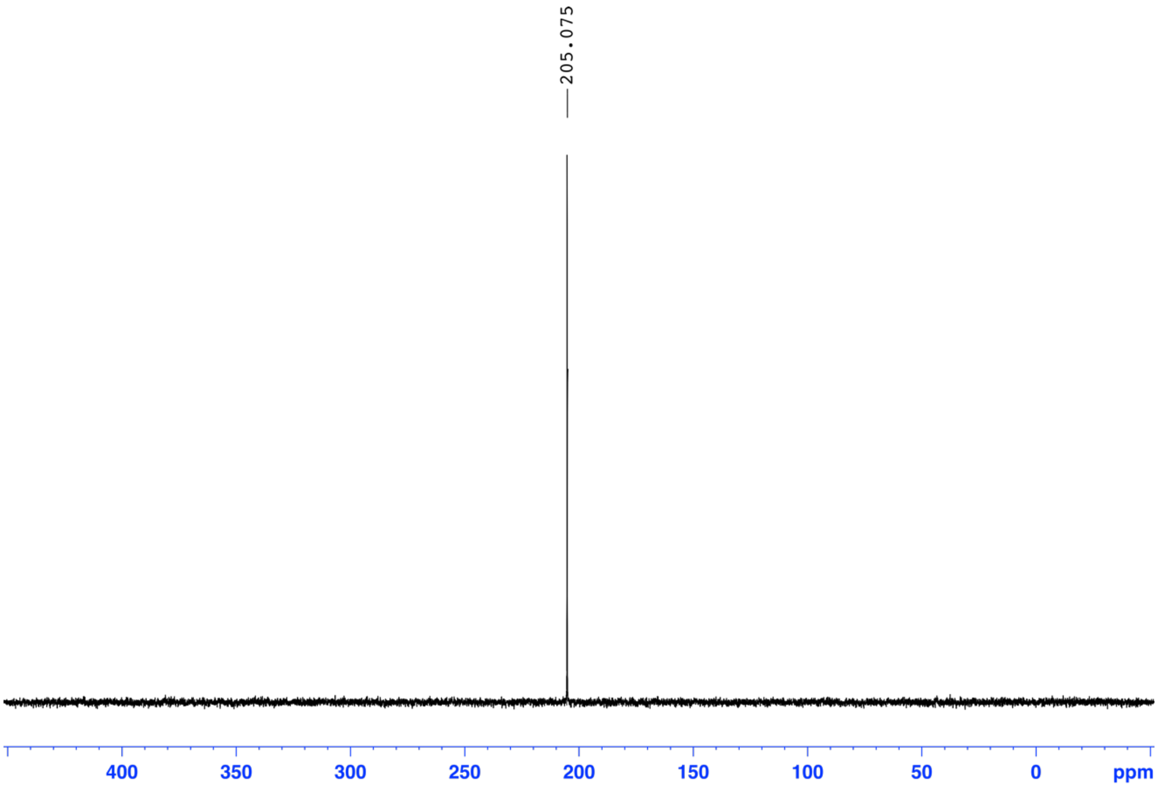

Figure S-11. The ^{31}P NMR spectrum of [(POBBOP)(Ru)(CO)$_2$(Au)(Cl)]$_2$ (**2-Au**) in CD_2Cl_2.

^{31}P{^{1}H} (CD_2Cl_2): δ 205.1

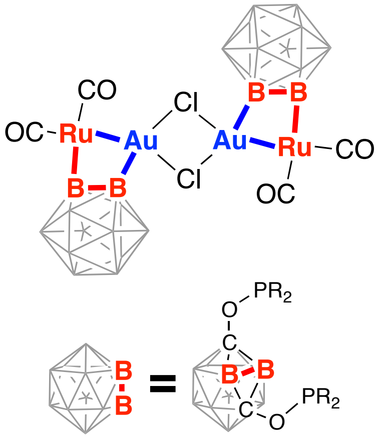

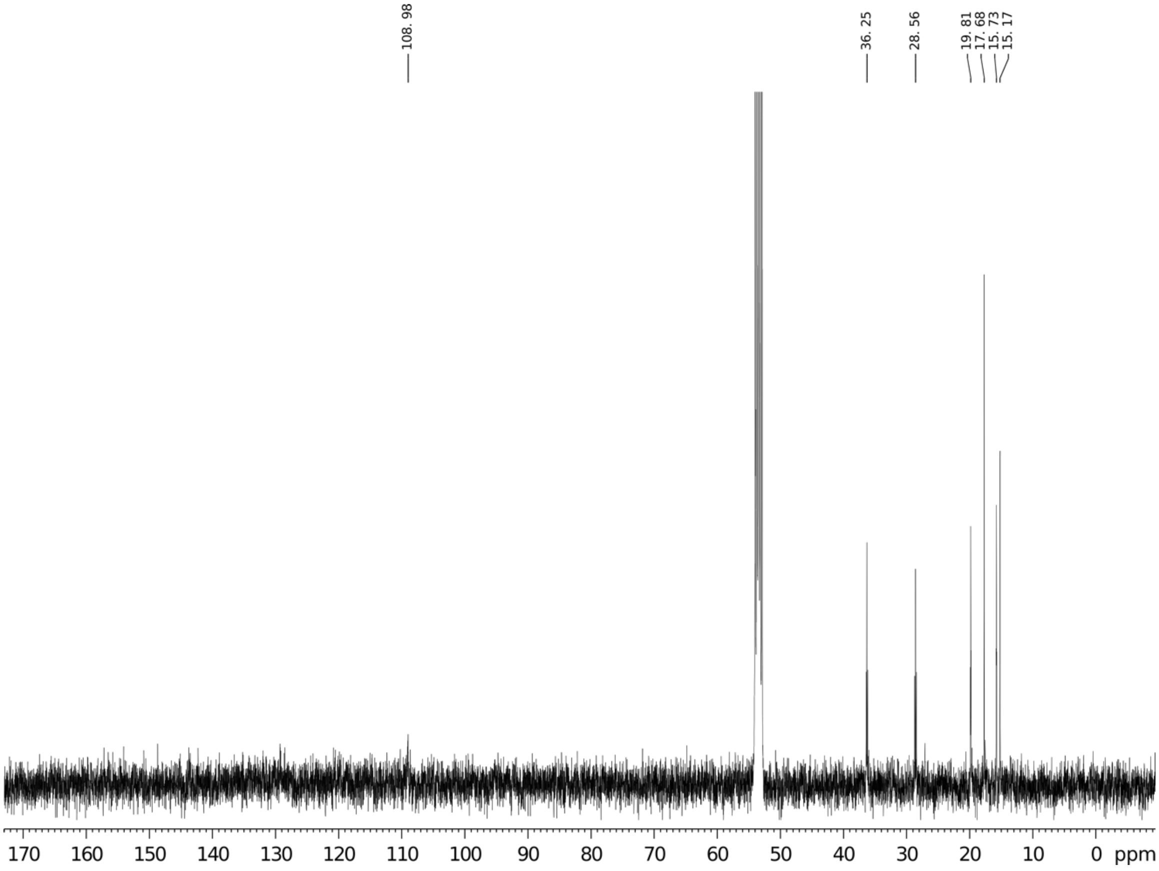

Figure S-12. The ^{13}C NMR spectrum of $[(POBBOP)(Ru)(CO)_2(Au)(Cl)]_2$ (**2-Au**) in CD_2Cl_2.

^{13}C (CD_2Cl_2): δ 109.0 ($C_2B_{10}H_{10}$), 36.3 (P*C*H(CH_3)$_2$), 28.6 (P*C*H(CH_3)$_2$), 19.8 (PCH(CH$_3$)$_2$), 17.7 (PCH(CH$_3$)$_2$), 15.7 (PCH(CH$_3$)$_2$), 15.2 (PCH(CH$_3$)$_2$)

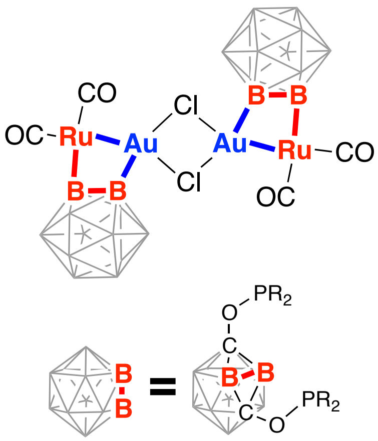

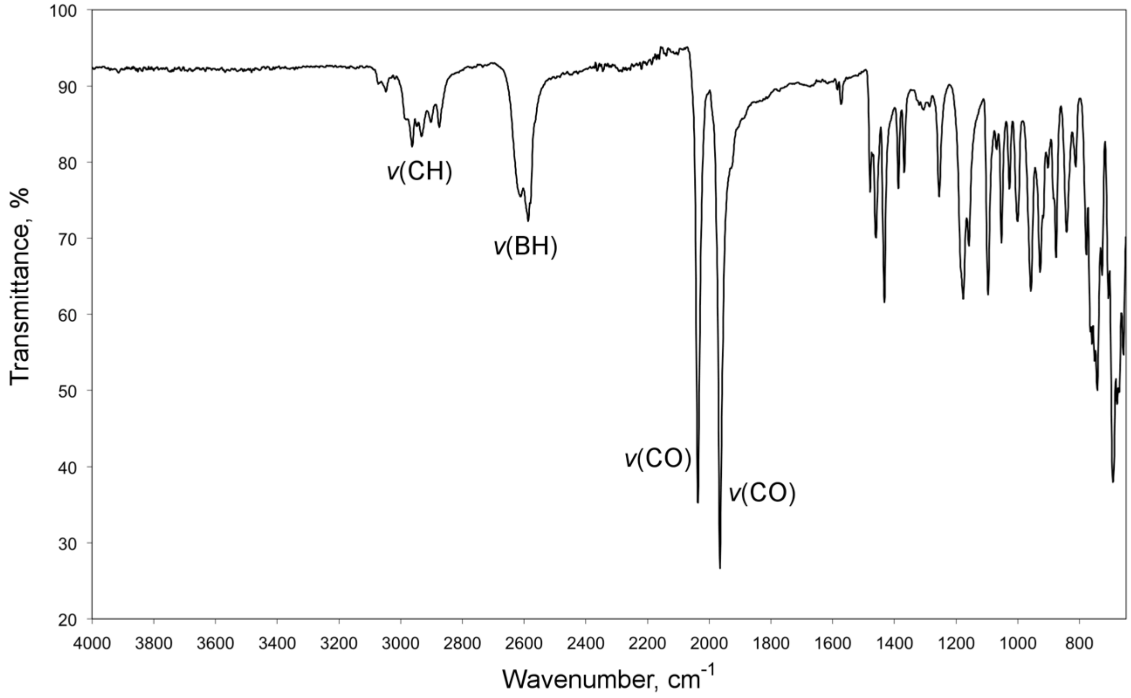

Figure S-13. The ATR-FTIR absorption spectrum of a solid sample of $[(POBBOP)(Ru)(CO)_2(Au)(Cl)]_2$ (**2-Au**).

Selected bands in the IR spectrum, cm^{-1}: 2992–2878 (br, CH), 2590 (br, BH), 2038 (strong, Ru-*CO*), 1966 (strong, Ru-*CO*).

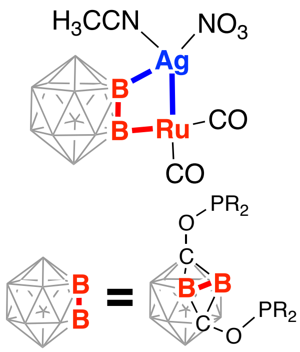

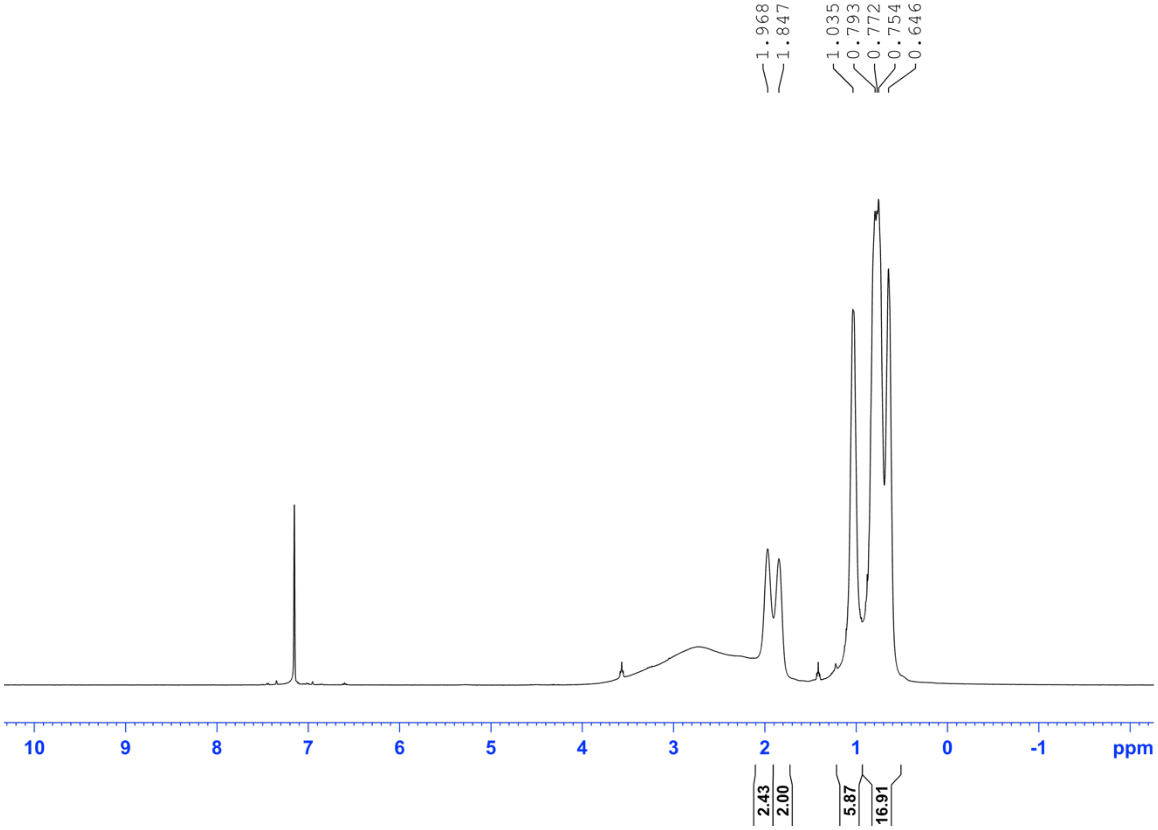

Figure S-14. The ^{1}H NMR spectrum of (POBBOP)(Ru)$(CO)_2$(Ag)$(CH_3CN)(NO_3)$ (**2-Ag**) in C_6D_6.

^{1}H NMR (C_6D_6): δ 1.97 (br, 2H, (PC$H$$(CH_3)_2$), 1.85 (br, 2H, (PC$H$$(CH_3)_2$), 1.04 (m, 6H, (PCH(C$H_3$$)_2$), 0.78 (m, 12H, (PCH(C$H_3$$)_2$), 0.65 (m, 6H, (PCH(C$H_3$$)_2$).

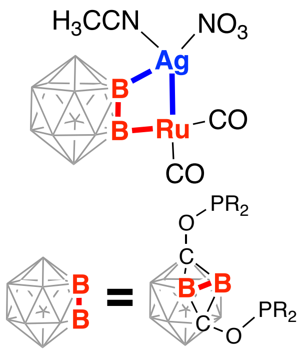

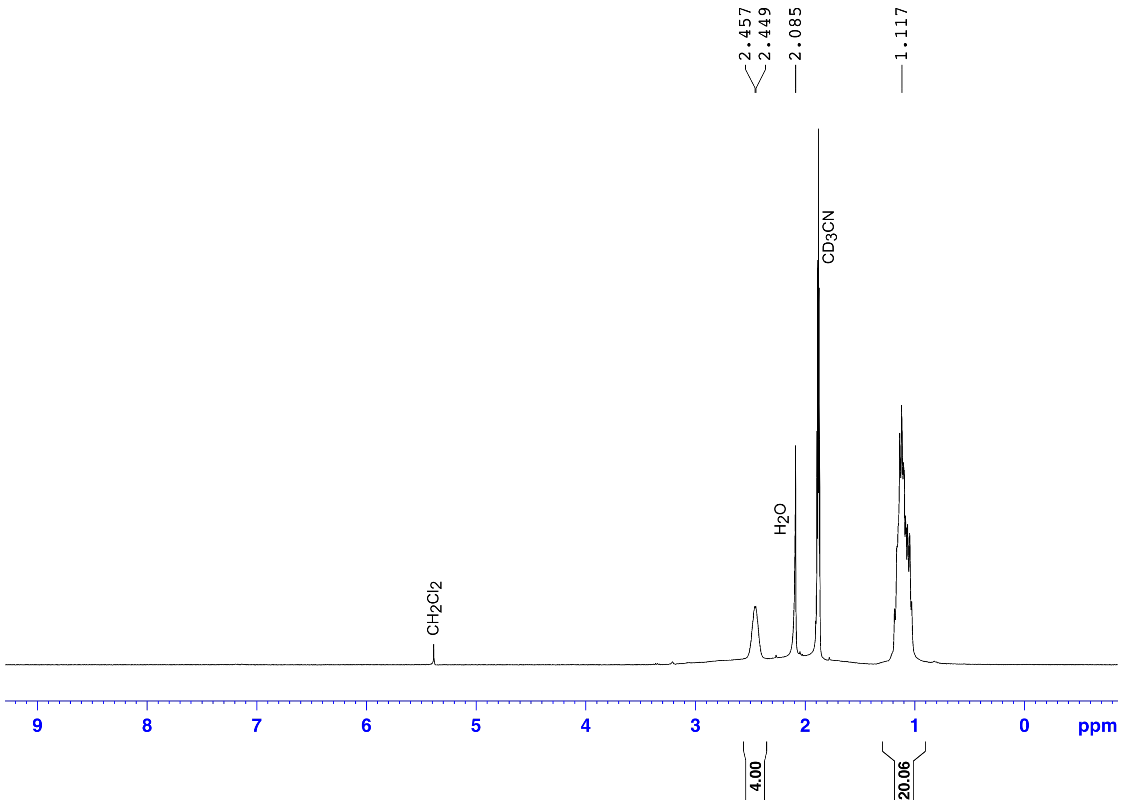

Figure S-14A. The ^{1}H NMR spectrum of (POBBOP)(Ru)$(CO)_2$(Ag)(CH_3CN)(NO_3) (**2-Ag**) in CD_3CN.

^{1}H NMR (CD_3CN): δ 2.45 (br, 4H, (PC*H*$(CH_3)_2$), 1.12 (m, 24H, (PCH(C*H*$_3$)$_2$).

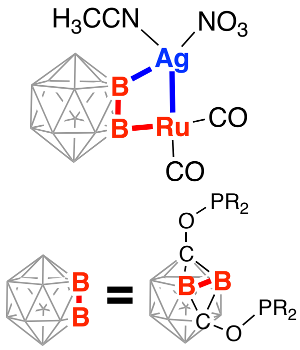

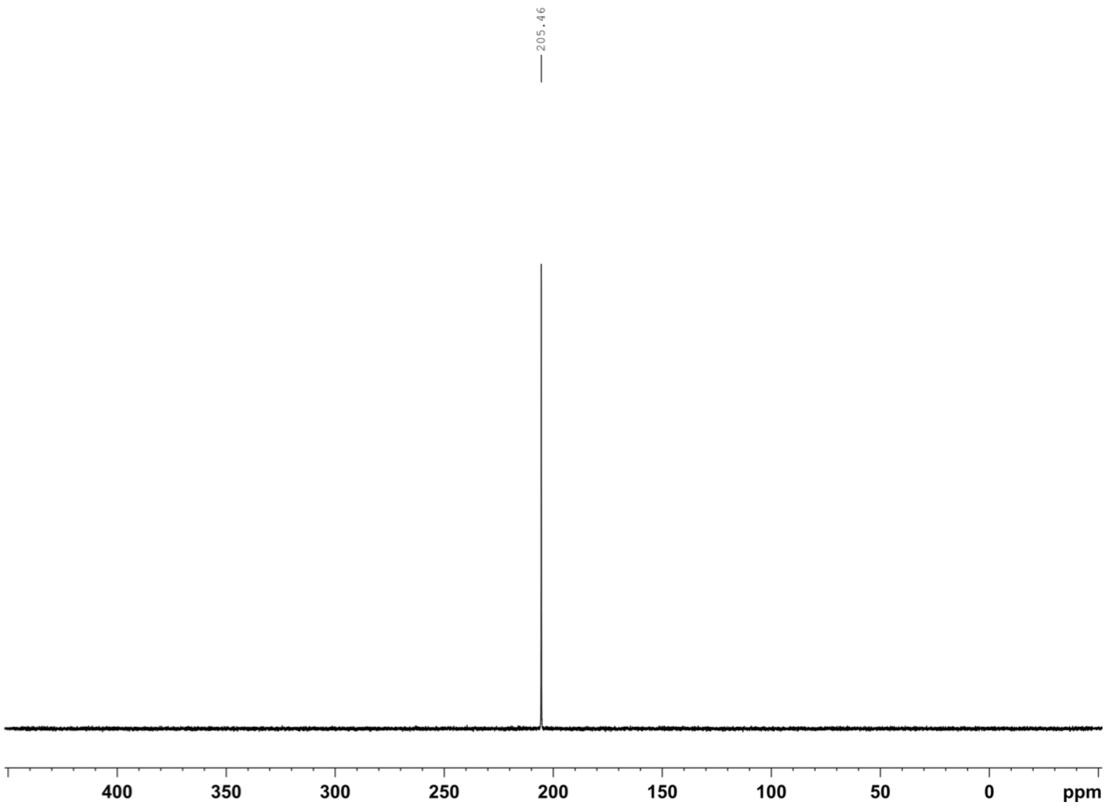

Figure S-15. The ^{31}P NMR spectrum of (POBBOP)(Ru)$(CO)_2$(Ag)(CH_3CN)(NO_3) (**2-Ag**) in C_6D_6.

$^{31}P\{^1H\}$ (C_6D_6): δ 205.5.

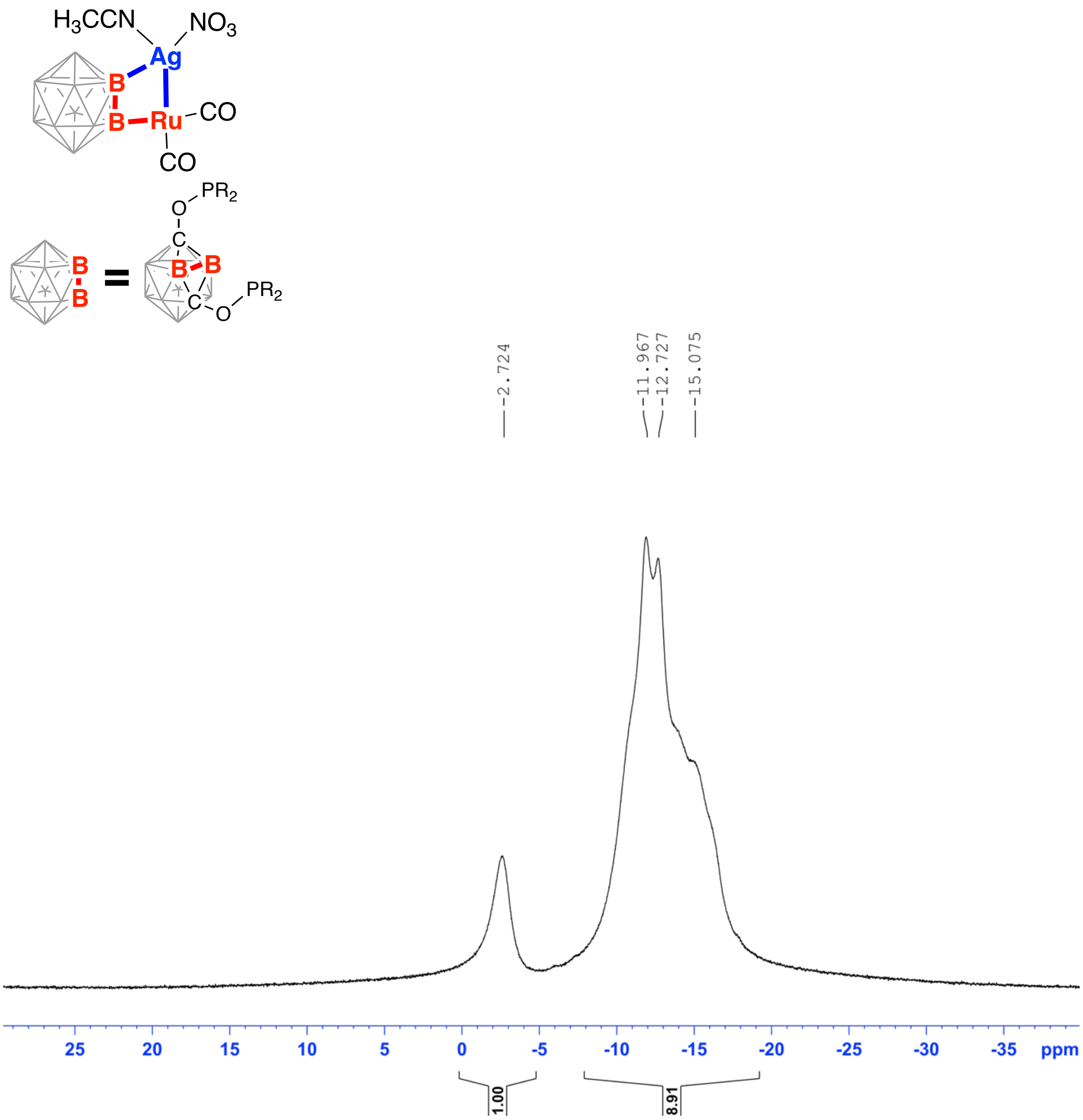

Figure S-16. The ^{11}B NMR spectrum of (POBBOP)(Ru)$(CO)_2$(Ag)$(CH_3CN)(NO_3)$ (**2-Ag**) in C_6D_6.

[11]B{[1]H} (C_6D_6): δ -2.7 (*B*-Ru), -11.9 (*B*-Ag), -12.8 (*B*-H), -15.0 (*B*-H), -15.4 (*B*-H)

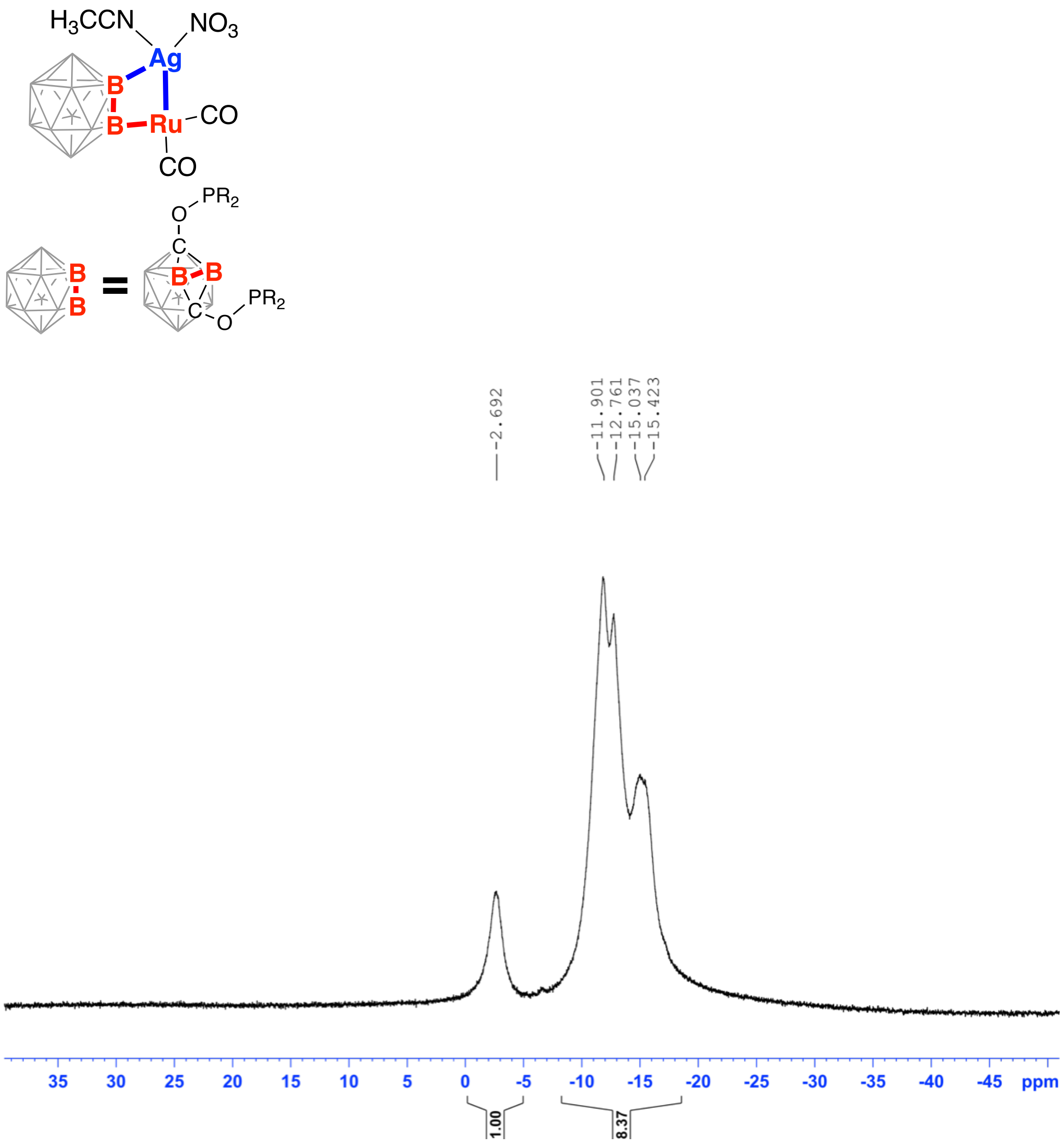

Figure S-17. The $^{11}B\{^{1}H\}$ NMR spectrum of (POBBOP)(Ru)$(CO)_2$(Ag)(CH_3CN)(NO_3) (**2-Ag**) in C_6D_6.

^{11}B{^{1}H} (C_6D_6): δ -2.7 (*B*-Ru), -11.9 (*B*-Ag), -12.8 (*B*-H), -15.0 (*B*-H), -15.4 (*B*-H)

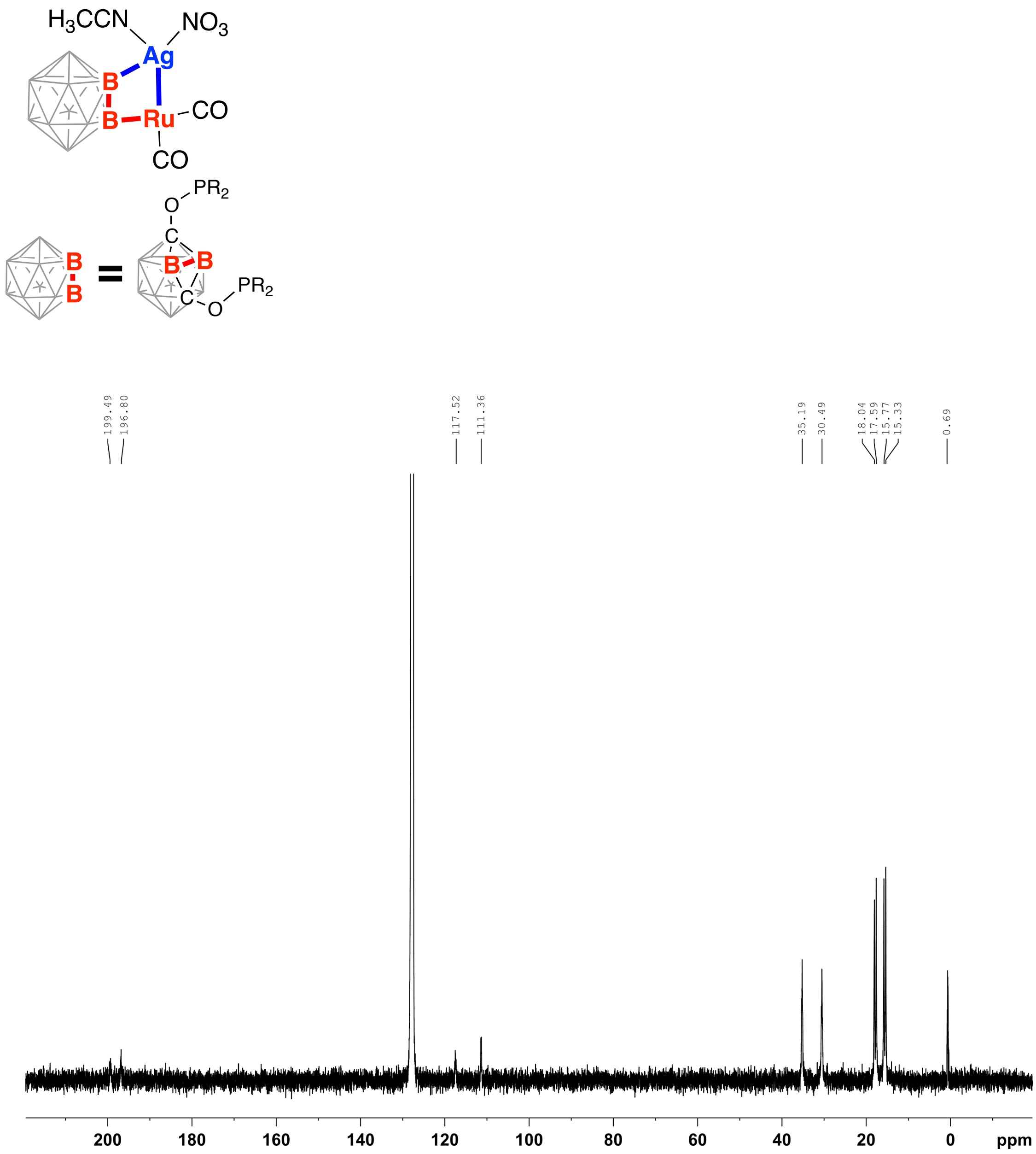

Figure S-18. The ^{13}C NMR spectrum of (POBBOP)(Ru)$(CO)_2$(Ag)(CH_3CN)(NO_3) (**2-Ag**) in C_6D_6.

^{13}C (C_6D_6): δ 199.5 (Ru-*C*O), 196.8 (Ru-*C*O), 117.5 (CH_3*C*N), 111.4 ($C_2B_{10}H_{10}$), 35.2 (P*C*H(CH_3)$_2$), 30.5 (P*C*H(CH_3)$_2$), 17.8 (PCH(*C*H_3)$_2$), 15.5 (PCH(*C*H_3)$_2$), 0.7 (*C*H_3CN).

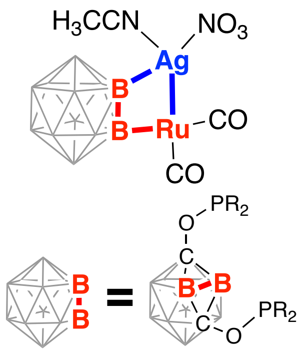

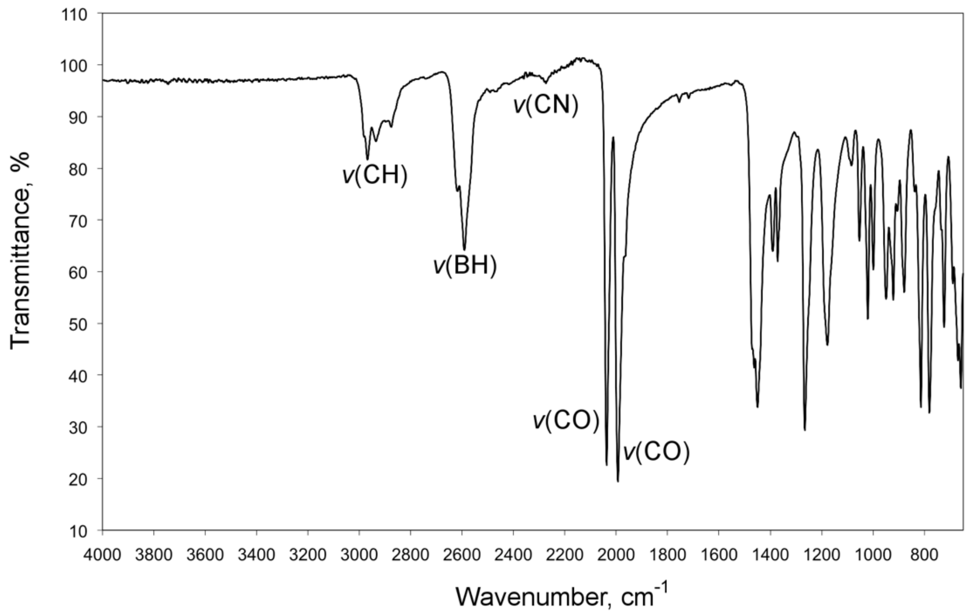

Figure S-19. The ATR-FTIR absorption spectrum of a solid sample of (POBBOP)(Ru)$(CO)_2$(Ag)$(CH_3CN)(NO_3)$ (**2-Ag**).

Selected bands in the IR spectrum, cm^{-1}: 2971–2882 (br, *CH*), 2594 (br, *BH*), 2283 (br, Ru-*NC*CH_3), 2038 (strong, Ru-*CO*), 1995 (strong, Ru-*CO*).

Theoretical Calculations.

For the topological analysis of the electron density with the QTAIM approach, the structures were optimized at the PBE0/def2-TZVP approach with ZORA correction as implemented in Orca 3.0.3 suite.[1,2] QTAIM analysis was then performed with the use of AIMAll code,[3] Laplacian maps were visualized with Multiwfn 3.3.9 software,[4] ELF functions were computed using TopMod 09 package.[5,6] The optimized structures were found to be in a good agreement with crystal structures for the bimetallic complexes. The optimized model of the dimer **2-Cu** had a distortion in coordination of bridging chloride ligands that did not affect the rest of the complex including bond distances and angles for the B1–Ru1–Cu1–B2 fragment.

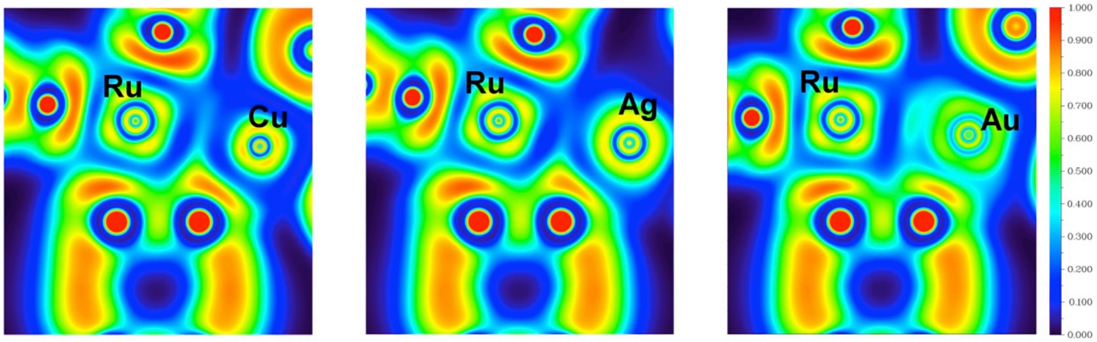

Figure S-20. ELF maps near the Ru–M–B2 fragment plotted in the Ru–B1–B2 plane.

Table S-1. ELF analysis of the bonding in the B1-Ru-M-B2 fragment. The values in the table are attractor values (η_a; the maximum ELF values for each basin) and basin populations (Ω, the number of electron per basin): η_a/Ω

	**2-Cu**	**2-Ag**	**2-Au**
V(Ru,B1)	0.899/1.67; 0.901/1.67	0.905/1.68	0.896/1.75; 0.896/1.74
V(Ru,M,B2)	0.863/2.57; 0.865/2.57	0.845/2.00	
V(Au,B2)			0.833/1.93; 0.833/1.94
V(Ru,Au)			0.431/0.71; 0.431/0.72
V(B1,B2)	0.699/0.36; 0.699/0.37	0.705/0.41	0.703/0.23; 0.703/0.23

half of the Ω value can be tentatively correlated with the bond order

References.

1. F. Neese, *WIREs Comput. Mol. Sci.*, 2012, **2**, 73-78.
2. D. A. Pantazis, X.-Y. Chen, C. R. Landis and F. Neese, *J. Chem. Theory Comput.*, 2008, **4**, 908-919.
3. T. A. Keith, in *AIMAll (Version 14.04.17), http://aim.tkgristmill.com*, 2014.
4. T. Lu and F. Chen, *J. Comput. Chem.*, 2012, **33**, 580-592.
5. Noury, S.; Krokidis, X.; Fuster, F.; Silvi, B., Computational tools for the electron localization function topological analysis. *Computers and Chemistry* 1999, 23, 597-604.
6. Noury, S.; Krokidis, X.; Fuster, F.; Silvi, B. TopMoD 09 package, 2009. http://www.lct.jussieu.fr/article76.html.

X-Ray Structure Determination, [(POBBOP)(Ru)$(CO)_2$(Cu)(Cl)$]_2$ (2-Cu)

X-ray intensity data from a colorless block crystal were collected at 100(2) K using a Bruker D8 QUEST diffractometer equipped with a PHOTON 100 CMOS area detector and an Incoatec microfocus source (Mo Kα radiation, λ = 0.71073 Å).[1] The raw area detector data frames were reduced and corrected for absorption effects using the SAINT+ and SADABS programs.[1] Final unit cell parameters were determined by least-squares refinement of 9926 reflections taken from the data set. The structure was solved by direct methods with SHELXT.[2] Subsequent difference Fourier calculations and full-matrix least-squares refinement against F^2 were performed with SHELXL-2014[2] using OLEX2.[3]

The compound crystallizes in the monoclinic system. The pattern of systematic absences in the intensity data was consistent with the space group $P2_1/n$, which was confirmed by structure solution. The asymmetric unit consists of half of one $\{Ru(CO)_2(C_{14}H_{36}B_{10}O_2P_2)\}_2(CuCl)_2$ complex, which is located on a crystallographic inversion center. All non-hydrogen atoms were refined with anisotropic displacement parameters. Hydrogen atoms bonded to carbon were located in Fourier difference maps before being placed in geometrically idealized positions included as riding atoms with *d*(C-H) = 1.00 Å and Uiso(H) = 1.2Ueq(C) for methine hydrogen atoms and *d*(C-H) = 0.98 Å and Uiso(H) = 1.5Ueq(C) for methyl hydrogens. The methyl hydrogens were allowed to rotate as a rigid group to the orientation of maximum observed electron density. Hydrogen atoms bonded to boron were located in difference maps and refined freely. The largest residual electron density peak in the final difference map is 0.47 e-/Å^3 located 0.91 Å from P2.

(1) APEX2 Version 2014.9-0, SAINT+ Version 8.34A and SADABS Version 2014/4. Bruker Analytical X-ray Systems, Inc., Madison, Wisconsin, USA, 2014.

(2) (a) **SHELXT**: Sheldrick, G.M. *Acta Cryst.* **2015**, *A71*, 3-8. (b) **SHELXL:** Sheldrick, G.M. *Acta Cryst.* **2008**, A64, 112-122.

(3) Dolomanov, O. V., Bourhis, L. J., Gildea, R. J., Howard J. A. K. and Puschmann, H. OLEX2: a complete structure solution, refinement and analysis program. *J. Appl. Cryst.* **2009**, *42*, 339-341.

Table S-2. Crystal data and structure refinement for $[(POBBOP)(Ru)(CO)_2(Cu)(Cl)]_2$ (**2-Cu**)

Identification code	BE366b
Empirical formula	$C_{32}H_{72}B_{20}Cl_2Cu_2O_8P_4Ru_2$
Formula weight	1325.09
Temperature/K	100(2)
Crystal system	monoclinic
Space group	$P2_1/n$
a/Å	10.4786(5)
b/Å	16.1010(8)
c/Å	16.7081(8)
α/°	90
β/°	92.821(2)
γ/°	90
Volume/Å^3	2815.5(2)
Z	2
ρ_{calc}g/cm^3	1.563
μ/mm^{-1}	1.523
F(000)	1336.0
Crystal size/mm^3	0.24 × 0.2 × 0.18
Radiation	MoKα (λ = 0.71073)
2Θ range for data collection/°	4.492 to 56.676
Index ranges	$-13 \le h \le 13$, $-21 \le k \le 21$, $-22 \le l \le 22$
Reflections collected	71901
Independent reflections	6999 [R_{int} = 0.0548, R_{sigma} = 0.0348]
Data/restraints/parameters	6999/0/357
Goodness-of-fit on F^2	1.041
Final R indexes [I>=2σ (I)]	R_1 = 0.0284, wR_2 = 0.0472
Final R indexes [all data]	R_1 = 0.0414, wR_2 = 0.0501
Largest diff. peak/hole / e Å^{-3}	0.47/-0.44

X-Ray Structure Determination, [(POBBOP)(Ru)(CO)$_2$(Au)(Cl)]$_2$ (2-Au)

X-ray intensity data from a colorless plate were collected at 100(2) K using a Bruker D8 QUEST diffractometer equipped with a PHOTON 100 CMOS area detector and an Incoatec microfocus source (Mo Kα radiation, λ = 0.71073 Å).[1] The raw area detector data frames were reduced and corrected for absorption effects using the SAINT+ and SADABS programs.[1] Final unit cell parameters were determined by least-squares refinement of 9793 reflections taken from the data set. The structure was solved by direct methods with SHELXT.[2] Subsequent difference Fourier calculations and full-matrix least-squares refinement against F^2 were performed with SHELXL-2014[2] using OLEX2.[3]

The compound crystallizes in the monoclinic system. The pattern of systematic absences in the intensity data was consistent with the space group $P2_1/c$, which was confirmed by structure solution. The asymmetric unit consists of half of one $\{Ru(CO)_2(C_{14}H_{36}B_{10}O_2P_2)\}_2(AuCl)_2$ complex, which is located on a crystallographic inversion center. All non-hydrogen atoms were refined with anisotropic displacement parameters. Hydrogen atoms bonded to carbon were located in Fourier difference maps before being placed in geometrically idealized positions and included as riding atoms with d(C-H) = 1.00 Å and Uiso(H) = 1.2Ueq(C) for methine hydrogen atoms and d(C-H) = 0.98 Å and Uiso(H) = 1.5Ueq(C) for methyl hydrogens. The methyl hydrogens were allowed to rotate as a rigid group to the orientation of maximum observed electron density. Hydrogen atoms bonded to boron were located in difference maps and refined freely. The largest residual electron density peak in the final difference map is 0.66 $e^-/Å^3$, located 0.07 Å from Au1.

(1) APEX2 Version 2014.9-0, SAINT+ Version 8.34A and SADABS Version 2014/4. Bruker Analytical X-ray Systems, Inc., Madison, Wisconsin, USA, 2014.

(2) (a) **SHELXT**: Sheldrick, G.M. *Acta Cryst.* **2015**, *A71*, 3-8. (b) **SHELXL:** Sheldrick, G.M. *Acta Cryst.* **2008**, A64, 112-122.

(3) Dolomanov, O. V., Bourhis, L. J., Gildea, R. J., Howard J. A. K. and Puschmann, H. OLEX2: a complete structure solution, refinement and analysis program. *J. Appl. Cryst.* **2009**, *42*, 339-341.

Table S-3. Crystal data and structure refinement for $[(POBBOP)(Ru)(CO)_2(Au)(Cl)]_2$ (**2-Au**)

Identification code	BE395
Empirical formula	$C_{32}H_{72}Au_2B_{20}Cl_2O_8P_4Ru_2$
Formula weight	1591.94
Temperature/K	100(2)
Crystal system	monoclinic
Space group	$P2_1/c$
a/Å	12.4210(5)
b/Å	12.5960(6)
c/Å	19.0782(8)
α/°	90
β/°	101.457(2)
γ/°	90
Volume/Å^3	2925.4(2)
Z	2
ρ_{calc}g/cm^3	1.807
μ/mm^{-1}	5.748
F(000)	1536.0
Crystal size/mm^3	0.18 × 0.16 × 0.04
Radiation	MoKα (λ = 0.71073)
2Θ range for data collection/°	4.356 to 60.268
Index ranges	$-17 \leq h \leq 17$, $-17 \leq k \leq 17$, $-26 \leq l \leq 26$
Reflections collected	155104
Independent reflections	8607 [R_{int} = 0.0558, R_{sigma} = 0.0234]
Data/restraints/parameters	8607/0/357
Goodness-of-fit on F^2	1.079
Final R indexes [I>=2σ (I)]	R_1 = 0.0225, wR_2 = 0.0340
Final R indexes [all data]	R_1 = 0.0301, wR_2 = 0.0352
Largest diff. peak/hole / e Å^{-3}	0.67/-0.74

X-Ray Structure Determination, (POBBOP)(Ru)(Ag)(CH_3CN)(NO_3) (2-Ag)

X-ray intensity data from a colorless prism were collected at 100(2) K using a Bruker D8 QUEST diffractometer equipped with a PHOTON-100 CMOS area detector and an Incoatec microfocus source (Mo Kα radiation, λ = 0.71073 Å). The raw area detector data frames were reduced and corrected for absorption effects using the Bruker APEX3, SAINT+ and SADABS programs.[1,2] Final unit cell parameters were determined by least-squares refinement of 9913 reflections taken from the data set. The structure was solved by direct methods with SHELXT.[3] Subsequent difference Fourier calculations and full-matrix least-squares refinement against F^2 were performed with SHELXL-2014[3] using OLEX2.[4]

The compound crystallizes in the monoclinic system. The pattern of systematic absences in the intensity data was consistent with the space groups *P*2/*n* and *Pn*. The acentric group *Pn* was confirmed by structure solution and by analysis with ADDSYM,[5] which found no missed symmetry. The asymmetric unit consists of one molecule. All non-hydrogen atoms were refined with anisotropic displacement parameters. Hydrogen atoms bonded to carbon were located in Fourier difference maps before being placed in geometrically idealized positions and included as riding atoms with *d*(C-H) = 1.00 Å and *U*iso(H) = 1.2*U*eq(C) for methine hydrogen atoms and *d*(C-H) = 0.98 Å and *U*iso(H) = 1.5*U*eq(C) for methyl hydrogens. The methyl hydrogens were allowed to rotate as a rigid group to the orientation of maximum observed electron density. Hydrogen atoms bonded to boron were located in difference maps and refined freely. The largest residual electron density peak in the final difference map is 0.33 $e^-/Å^3$, located 0.69 Å from C2. The absolute structure (Flack) parameter after the final refinement cycle was 0.003(7).

(1) **APEX3** Version 2016.5-0 and **SAINT+** Version 8.37A. Bruker AXS, Inc., Madison, Wisconsin, USA, 2016.

(2) **SADABS**-2016/2: Krause, L., Herbst-Irmer, R., Sheldrick G.M. and Stalke D. *J. Appl. Cryst.* **2015**, *48*, 3-10.

(3) (a) **SHELXT**: Sheldrick, G.M. *Acta Cryst.* **2015**, *A71*, 3-8. (b) **SHELXL:** Sheldrick, G.M. *Acta Cryst.* **2008**, A64, 112-122.

(4) **OLEX2**: a complete structure solution, refinement and analysis program. Dolomanov, O. V., Bourhis, L. J., Gildea, R. J., Howard J. A. K. and Puschmann, H. *J. Appl. Cryst.* **2009**, *42*, 339-341.

(5) (a) LePage, Y. *J. Appl. Crystallogr.* **1987**, *20*, 264-269. (b) Spek, A. L. *J. Appl. Crystallogr.*, **1988**, *21*, 578-579. (c) Spek, A. L. *Acta Crystallogr., Sect A* **1990**, *46*, C34. (d) PLATON: Spek, A.L. *Acta Cryst.,* **2009**, *D65*, 148-155.

Table S-4. Crystal data and structure refinement for **(POBBOP)(Ru)(Ag)(CH_3CN)(NO_3) (2-Ag)**

Identification code	BE3166
Empirical formula	$C_{18}H_{39}AgB_{10}N_2O_7P_2Ru$
Formula weight	774.49
Temperature/K	100(2)
Crystal system	monoclinic
Space group	Pn
a/Å	10.6009(5)
b/Å	13.8331(7)
c/Å	11.0738(5)
α/°	90
β/°	103.867(2)
γ/°	90
Volume/Å^3	1576.57(13)
Z	2
ρ_{calc}g/cm^3	1.631
μ/mm^{-1}	1.241
F(000)	776.0
Crystal size/mm^3	0.44 × 0.38 × 0.22
Radiation	MoKα (λ = 0.71073)
2Θ range for data collection/°	5.614 to 65.344
Index ranges	$-16 \leq h \leq 15, -21 \leq k \leq 20, -16 \leq l \leq 16$
Reflections collected	50528
Independent reflections	11466 [R_{int} = 0.0384, R_{sigma} = 0.0373]
Data/restraints/parameters	11466/2/412
Goodness-of-fit on F^2	1.031
Final R indexes [I>=2σ (I)]	R_1 = 0.0215, wR_2 = 0.0379
Final R indexes [all data]	R_1 = 0.0250, wR_2 = 0.0387
Largest diff. peak/hole / e Å^{-3}	0.33/-0.34
Flack parameter	0.003(7)

(a)

(b)

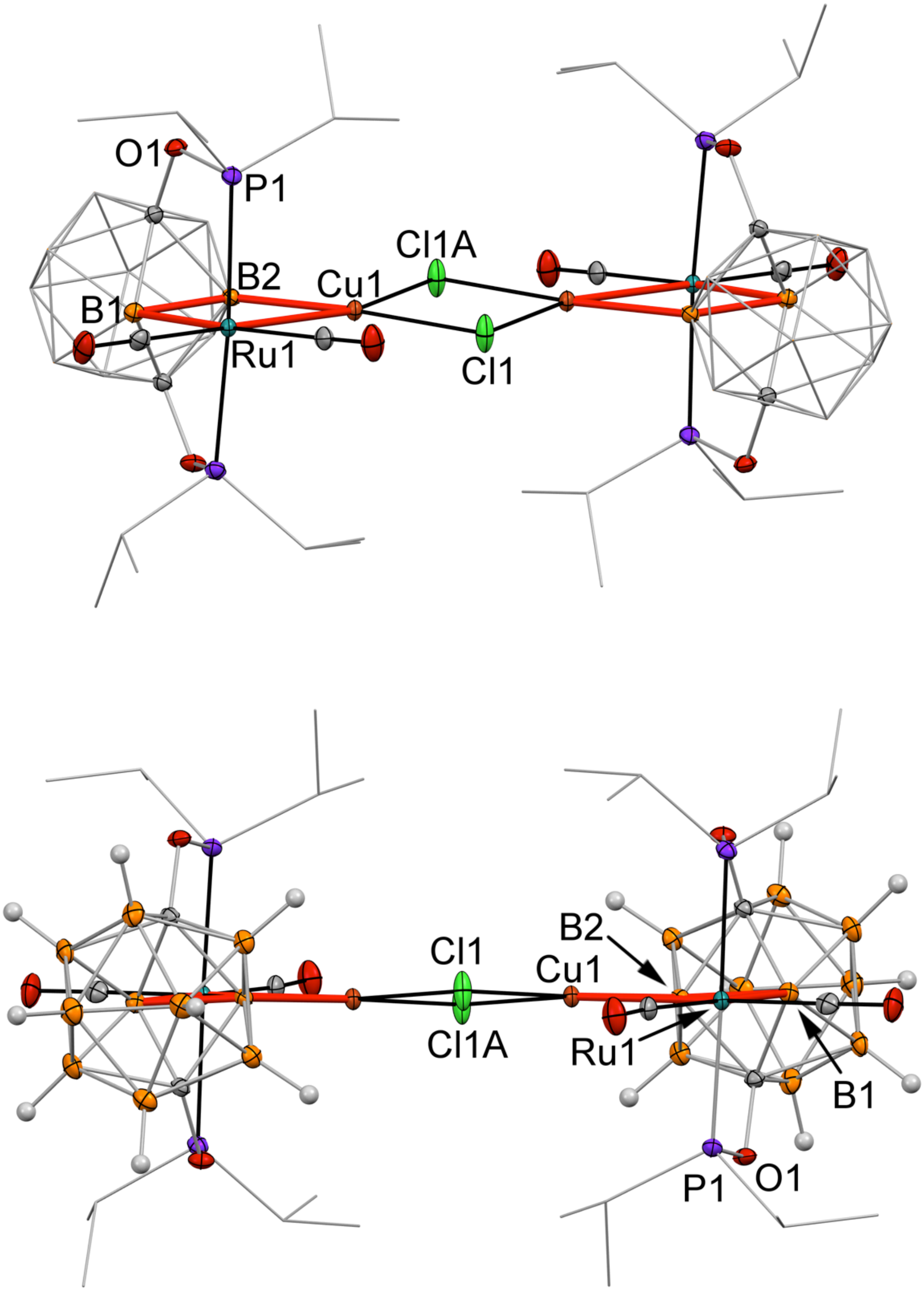

Figure S-21. Displacement ellipsoid plot (50% probability) of the $[(POBBOP)Ru(CO)_2(Cu)(Cl)]_2$ complex (**2-Cu**). (a): a general view (b): a view parallel to the B2–Cu1–Ru1–B1 plane demonstrating planarity of the environment of the copper center. Hydrogen atoms, and the majority of boron and carbon atoms are omitted for clarity.

(a)

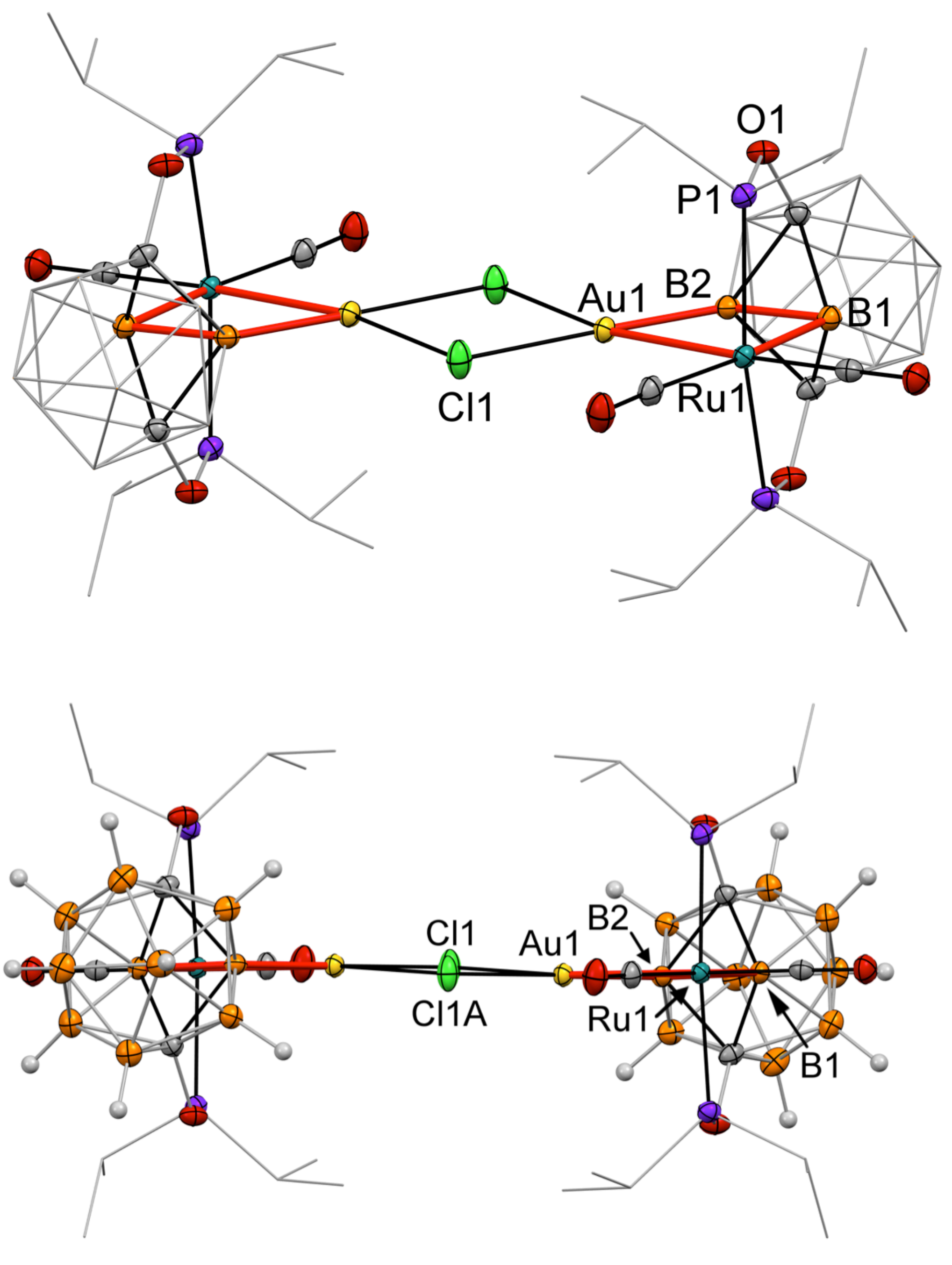

(b)

Figure S-22. Displacement ellipsoid plot (50% probability) of the $[(POBBOP)Ru(CO)_2(Au)(Cl)]_2$ complex (**2-Au**). (a): a general view (b): a view parallel to the B2–Au1–Ru1–B1 plane demonstrating planarity of the environment of the copper center. Hydrogen atoms, and the majority of boron and carbon atoms are omitted for clarity.

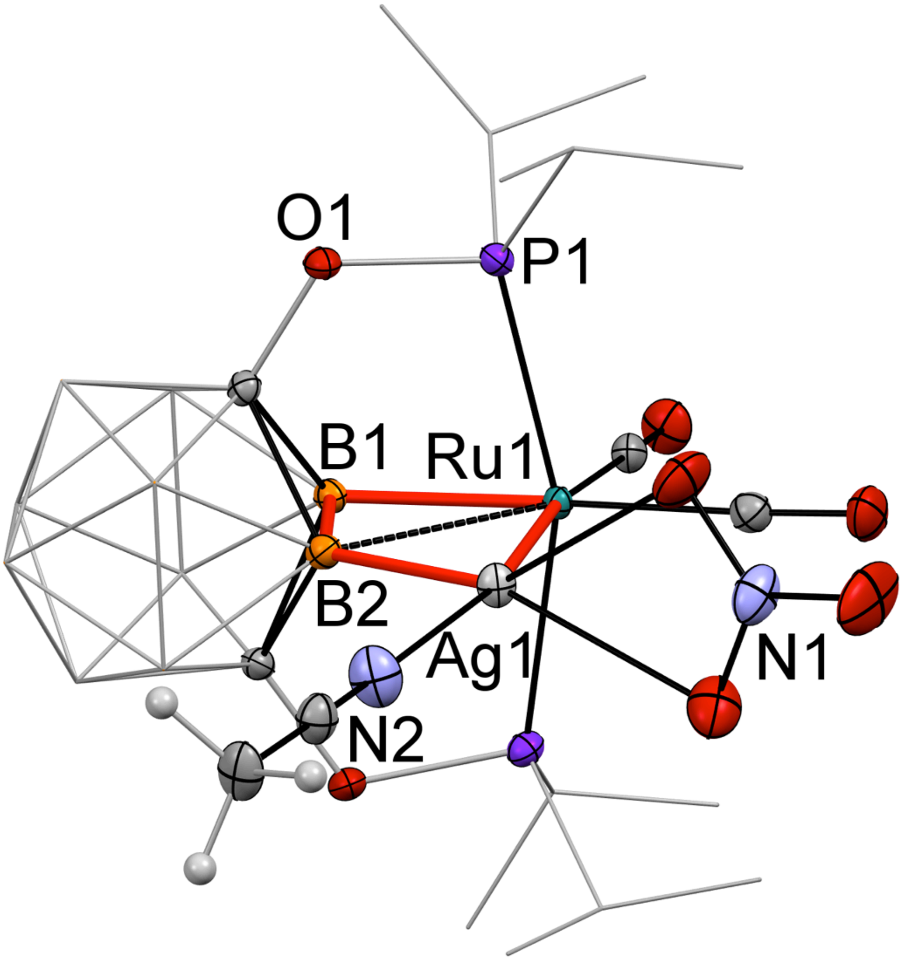

Figure S-23. Displacement ellipsoid plot (50% probability) of the [(POBBOP)Ru$(CO)_2$(Ag)(CH_3CN)(NO_3)] complex (**2-Ag**). A general view. Hydrogen atoms, and the majority of boron and carbon atoms are omitted for clarity.
